# Linking Acrosome Size and Genetic Divergence in an Inter-Oceanic Mussel from the Pacific and Atlantic Coasts: A Case of Incipient Speciation?

**DOI:** 10.3390/ani14050674

**Published:** 2024-02-21

**Authors:** Carolina Briones, José J. Nuñez, Montse Pérez, Orlando Garrido, Bernardita Campos, Karina Godoy, Ricardo Hartley, Pablo A. Oyarzún, Ricardo Guiñez

**Affiliations:** 1 Instituto de Ciencias Naturales Alexander von Humbodt, Facultad de Ciencias del Mar y de Recursos Biológicos, Universidad de Antofagasta, Angamos 601, Antofagasta 1270300, Chile; ricardo.guinez@uantof.cl; 2 Instituto de Ciencias Marinas y Limnológicas, Facultad de Ciencias, Universidad Austral de Chile, Casilla 567, Valdivia 5090000, Chile; jjnunez@uach.cl (J.J.N.); ogarrido@uach.cl (O.G.); 3 AquaCOV, Centro Oceanográfico de Vigo, Instituto Español de Oceanografía, Consejo Superior de Investigaciones Científicas (IEO, CSIC), 36390 Vigo, Spain; montse.perez@ieo.csic.es; 4 Independent Researcher, Orión 19, Viña del Mar 2580660, Chile; bernardita.campos@uv.cl; 5 Núcleo Científico y Tecnológico de Biorecursos (BIOREN), Universidad de La Frontera, Temuco 4811230, Chile; 6 Instituto de Investigación y Postgrado, Facultad de Medicina y Ciencias de la Salud, Universidad Central de Chile, Santiago 8330507, Chile; ricardo.hartley@ucentral.cl; 7 Centro de Investigación Marina Quintay (CIMARQ), Universidad Andrés Bello, Quintay 2340000, Chile

**Keywords:** bayesian method, DUI, ect-aquasperm, Last Glacial Maximum, Mytilidae, *Perumytilus purpuratus*, postglacial recolonization, reproductive isolation, sperm morphotypes

## Abstract

**Simple Summary:**

*Perumytilus purpuratus* is a mussel species that is broadly distributed along the latitudinal gradient from the Southern Pacific to the Atlantic Ocean. Along its distribution, *P. purpuratus* has been historically considered as one species. However, in the last decade, evidence has supported the hypothesis of two geographically divergent lineages. Within this context, we explore the evolutionary history of this inter-oceanic mussel, linking both sperm acrosome variability and molecular analyses of males at the geographic macroscale. In general, our results were in agreement with the genetic divergence that was previously reported, and showing two north and south lineage. Furthermore, our comprehensive sampling effort enabled us to precisely determine both the latitudinal positions of morpho-genetic break, which was identified at 37° S on the Pacific coast, and a probable hybridization zone at 38° S. In addition, we found new southern sperm variants, longer than previously reported. Overall, our results support the hypothesis of historical events and postglacial recolonization as causal phenomena for the observed divergences. Finally, we consider that, despite uncertainties in the taxonomy of *P. purpuratus*, the morpho-genetic divergence between the north and south *P. purpuratus* lineages indicates that they do not constitute the same Evolutionary Significant Unit and, therefore, they should be considered as separate entities.

**Abstract:**

In recent years, advances in analyses of the sperm morphology and genetics of *Perumytilus purpuratus* have allowed to two evolutionary scenarios for this mussel to be suggested: (1) the scenario of cryptic species and (2) the scenario of incipient or in progress speciation. For a better understanding of the evolutionary history of *P. purpuratus*, we performed extensive sampling along a latitudinal gradient of ca. 7180 km of coastline—from the Southern Pacific Ocean to the Atlantic Ocean—and we delved deeper into the sperm morphology of *P. purpuratus*, exploring its association with the phylogeny and population genetics to determine whether the variability in sperm traits between the northern and southern regions was a signal of cryptic or incipient species. Overall, our results showed that sperm sizes were strongly correlated with the genetic structure in males of *P. purpuratus*. We identified at 37° S on the Pacific coast a coincident break of both sperm size and genetic disruption that can be explained by historical events and postglacial recolonization as causal phenomena for the observed divergences. Furthermore, evidence of genetic admixture between lineages was found at 38° S, suggesting the presence of an introgressive hybridization zone and incomplete reproductive isolation in an *in fraganti* or incipient speciation process.

## 1. Introduction

Within an evolutionary context, both the taxonomic classification of organisms and the understanding of the diversity of biological life have historically been based on the description of morphological traits [1]. In fact, morphology is a very useful way of understanding evolutionary processes and has traditionally been used in systematics and taxonomy. In bivalve mollusks, such as clams [2], oysters [3,4], and mussels [5,6,7,8], sperm morphological traits have been used in systematic studies because the ultrastructure appears to be highly conserved at the species level, so it is used to differentiate closely related species [2]. In fact, due to this conservative aspect at the species level, the ultrastructure of sperm has been used for taxonomic purposes in several species (see [8]). We use the term “morphotype” in the sense of morphospecies [9] as an operational term that implies a distinct form (i.e., acrosome) of a given organism [9,10], which, as a working hypothesis, can be assigned to the category of species. We originally used this definition in Briones et al. (2012) [6]. Although the role of sperm morphology in fertilization is poorly understood, it is clear that spermatozoa vary widely in size, shape, and ornamentation between closely related species [11]. The interest in spermatozoa principally comes from their unusual life history and unparalleled diversity, which appear to be inextricably linked. Unique among metazoan cells, spermatozoa are cast forth from the soma into a foreign environment to spend their (pre-fertilization) lives as free-living organisms [12]. Evidence has demonstrated that sperm exhibit a remarkable diversity in form, mirroring the range of fertilization environments in nature [11].

In this work, we link acrosome morphotypes with genetic divergence at the geographic macroscale using the inter-oceanic mussel *Perumytilus purpuratus* (Lamarck 1819) as a model species. This species is broadly distributed on the Southeast Pacific coast, from Guayaquil (3° S) in Ecuador to Cabo de Hornos (53° S) in the continental Chilean coast [13], and it goes up through the Patagonia (41° S) on the South Atlantic coast of Argentina [14,15]. As in other externally fertilizing species of Mytilidae, the sperm of *P. purpuratus* are released into the water and are of the morphologically primitive or ect-aquasperm type, which is characterized by a conical or oval nucleus [5,6,16,17]; in addition, they have acrosomes of variable complexity, an intermediate piece composed of four or five mitochondria surrounding a pair of centrioles, and a simple flagellum [5,6,16]. Between the spermatic organelles, the acrosome appears to be the most interspecifically variable organelle, displaying diversity in shape and size [5,7,8,18], and it is the principal focus of many descriptive studies in this area.

Although the morphology and ultrastructure of sperm might provide useful traits for phylogenetic analyses [2,19,20], it is important to highlight that in the marine field, few studies have been conducted to explore the intraspecific variations in quantitative sperm traits and their associations with phylogeny. An exception was the comparative study conducted by Briones et al. (2012) [6] on *P. purpuratus* populations, where, based on acrosome size and sequence analyses of a mitochondrial rRNA gene (16S), two genetically divergent sperm acrosome morphotypes that were strongly related with their geographic origin were observed: a southern morphotype (previously described by Garrido and Gallardo (1996) [5] for Valdivia, 41° S) and a novel northern acrosome morphotype found at Valparaíso (32° S) and Antofagasta (23° S) [6]. The genetic divergence observed by these authors agreed with the findings of later studies at the geographical macroscale that confirmed a genetic discontinuity inside the distribution of *P. purpuratus*. On the one hand, based on mitochondrial (COI) and nuclear (18S and 28S) markers, Trovant et al. (2015) [21] observed two divergent lineages, which were designed as the North and South Clades. Using samples from the Pacific (Chile) and Atlantic (Argentina) coasts, these authors found that the samples between 20° S and 36° S on the Chilean coast corresponded to the North Clade, while those from localities south of 39° S on the Pacific coast (Chile) up to 42° S on the Atlantic coast (Argentina) corresponded to the South Clade. Alternatively, based on five polymorphic microsatellite markers, Guiñez et al. (2016) [22] determined two genetically divergent groups for *P. purpuratus* from the Pacific coast of Chile, and they designated them as stocks: a southern stock south of 40° S and a north-central stock between 18° S and 40° S. These authors suggested that this sharp structure was historical in nature and was maintained in *P. purpuratus* due to its environment-related restricted larval dispersal, its limited potential for dispersal, or a genetic/reproductive barrier between its historical lineages (see, e.g., [6,22]).

Overall, these findings suggest that the strong genetic break between the northern and southern regions of Chile could be a reflection of the postglacial colonization after the Last Glacial Maximum (LGM) [6,21,22] and that both morphological and genetical divergence inside the distributional range of *Perumytilus* (i.e., between the northern and southern populations) could, in fact, be evidence for two cryptic species [6,21,22].

Our aim was to deepen the study of the geographic variation in sperm morphology and their associations with phylogeny and population genetics to determine whether there is evidence of clinal variation in sperm traits of *P. purpuratus* or whether specimens from the northern and southern regions could correspond to cryptic species. In the last case, we expected that both sperm morphology and genetic patterns of discontinuity were strongly correlated.

To address these questions, we analyzed samples of *P. purpuratus* covering a coastal gradient of ca. 7180 km of coastline from the Pacific Ocean to the Atlantic Ocean. The sperm morphology was explored by using scanning electron microscopy (SEM), and statistically significant differences among the localities were explored with linear regression models. The genetic analyses were conducted using nuclear and paternal-inherited mitochondrial genes.

## 2. Materials and Methods

### 2.1. Sample Collection

**Figure 1:**
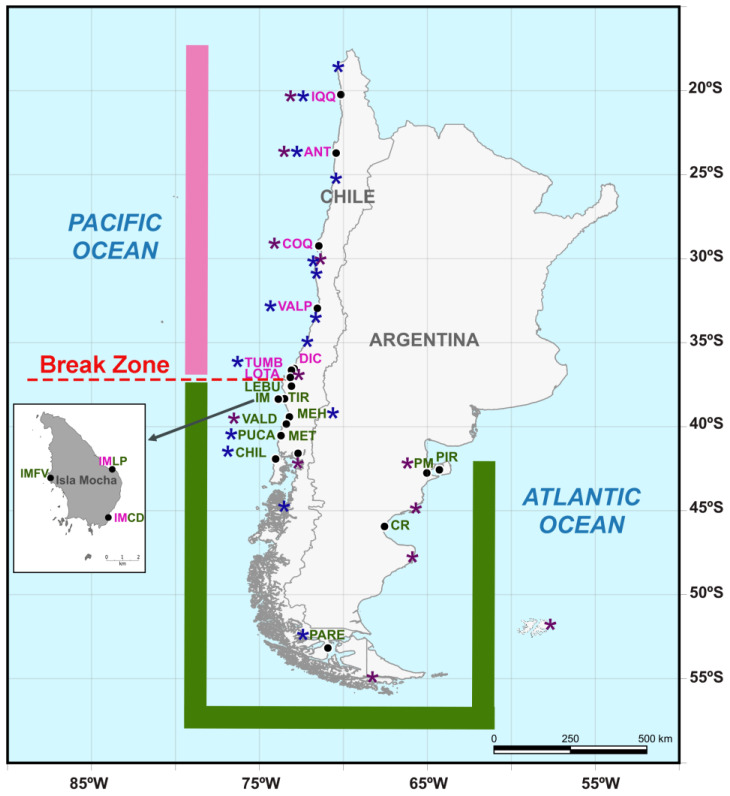
Map showing the continental sampling localities of *Perumytilus purpuratus*, which were distributed from 20° S on the Pacific coast of Chile to 42° S on the Argentinian Atlantic coast (black dots). Insular sampling localities (Isla Mocha) are displayed in the box. The distributional range and locality names for the north and south lineages are shown in pink and green colors, respectively. Localities in which both the north and south lineages were observed are shown with mixed colors (pink and green). The morpho-genetic break determined in this work is shown in red using a discontinuous line. To provide a complete picture of the known ranges of the two lineages, localities from Trovant et al. (2015) [21] and Guiñez et al. (2016) [22] are highlighted with purple and blue asterisks, respectively. The codes of the localities are shown in Table 1.

Freshly collected adults of *Perumytilus purpuratus*, with a maximum shell length of >15 mm, were obtained from mid-intertidal rocks along the Pacific and Atlantic coasts. All of the data used in this study were obtained from a total of 21 localities covering a coastal gradient of ca. 7180 km of coastline (Table 1, Figure 1), and they were sampled between 2010 and 2015.

### 2.2. Analyses of Sperm Morphology

This study was exclusively conducted on mature male specimens of *P. purpuratus*. Out of all of the localities encompassed within this study (i.e., 21), five were excluded from these analyses, as the individuals were not sexually mature at the time of sampling (see Table 1). Therefore, the morphological analyses were performed using a dataset of the quantitative sperm traits of a total of sixteen localities (Table 1, Figure 1). The sampled individuals were opened, and their sex and maturity were determined according to the color and thickness of the mantle tissue because in mature male individuals, it becomes thicker and turns into a cream color. Small pieces of the testes were stored in labelled microtubes, fixed in 2.5% glutaraldehyde in 0.2 M phosphate buffer (pH 7.4), and preserved at 4 °C until the scanning electron microscopy analyses. 

### 2.3. Scanning Electron Microscopy (SEM)

To capture the sperm morphological variability at the level of the locality and on a broad latitudinal scale, mixed sperm samples were used in the following scanning electron analyses.

For the samples obtained between 2010 and 2013, 100 µL of sperm suspensions from five selected individuals were mixed, and a drop of sperm suspension was placed on a cover glass, prefixed with 2.5% glutaraldehyde in a 0.2 M phosphate buffer (pH 7.4) for 2 h at 4 °C, and then post-fixed with 1% osmium tetroxide in a phosphate buffer for 2 h at 4 °C. The material was dehydrated in a graded ethanol series, dried to the critical point, coated with gold, and then observed and recorded with a Leo-420 scanning electron microscope.

For the sperm sampled in 2015, three individuals per locality were selected and fixed in 4% glutaraldehyde in a 0.2 M phosphate buffer (pH 7.4) with a volume of 400 µL and stored at a concentration of 6 × 10^7^ sperm/mL until analysis. Prior to the analysis, the sperm samples from each locality were mixed in a tube, washed twice with distilled water for 5 min, centrifuged at 1000 rpm, and filtered using a Nylon Membrane Millipore (40 µm). Subsequently, a droplet of sperm suspension was applied to double-faced aluminum adhesive tape affixed to an SEM stub. Visualization was performed using a secondary detector (SE) in a scanning electron microscope under the following conditions: 2.5 kV, working distance of 5.7 mm, and magnification of 6000× in Scanning SU 3500 HITACHI-Japan.

### 2.4. Statistical Analyses

From the scanning electron micrographs, the length of the acrosomes and heads of thirty spermatozoa per locality were measured using the Sigma Scan Pro v.5 and ImageJ programs while following Briones et al. (2012) [6]. Then, the acrosome/head ratio (%) was calculated, and basic descriptive statistics, such as the mean, standard deviation, and maximum and minimum values per locality, were estimated for all sperm parameters (acrosome, head, and the acrosome/head ratio (%)).

To determine the differences in the mean lengths of the acrosomes and heads among the localities, one-way ANOVA was performed with Locality as a fixed factor, followed by a post hoc Tukey’s test adjusted for multiple comparisons, using the PROC MIXED model (SAS). If the assumptions of the ANOVA were not met for the raw data, the rank-transformed data were used [23].

For the acrosome–head relationship, significant differences among localities were determined using a more robust statistical approximation instead of the acrosome/head ratio (%), which introduced biases into the statistical analyses, as stated by [24]. In this way, we performed an ANCOVA with Acrosome as the dependent variable and Head as a covariable (to control for size effects) while using Locality as a fixed factor. This represented the best-fitting, most parsimonious model using Akaike’s information criterion adjusted for small sample sizes (AICc) [25,26], which was previously selected from among the following candidate models: (1) the full model including Locality, Head, and the interaction term Head × Locality with slope heterogeneity among the localities; (2) reduced model 1 without the interaction term representing the homogeneity of slopes among localities; (3) reduced model 2 with only Locality as a covariable, implying that Head did not affect the acrosome length (which represented an ANOVA), and (4) reduced model 3, where only Head was the covariable but not Locality (which represented a single linear model). The model with the lowest AIC with Δ-AICc ≥ 2 was preferred, and at Δ-AICc < 2, the models were considered equivalent [27].

Then, a Tukey–Kramer post hoc test for multiple comparisons was applied using the adjusted acrosome length. The ANCOVA was run using PROC MIXED (SAS) independently for the northern and southern localities (i.e., north and south clades determined in the molecular analyses) given that the dependent variable (Acrosome) had a normal distribution within each lineage. 

For data exploration, statistical analysis, and graphing, Minitab® v 19.1 statistical software, SAS v 9.4/SAS Enterprise Guide 8.3 (Institute Inc., Cary, NC, USA), and OriginPro v 2023b SR1 (OriginLab Corporation, Northampton, MA, USA) were used. With SAS PROC UNIVARIATE, we tested the normal distributions of the dependent variables for the ANOVA and ANCOVA with a Kolmogorov–Smirnov test (K-S, *p* > 0.05).

### 2.5. Molecular Analyses

The genetic analyses were conducted at the geographic macroscale (ca. 7180 km from the Pacific to the Atlantic coast) to test hypotheses of genetic discontinuity [6,22,28] in relation to the geographically differentiated sperm (acrosome) morphotypes of *P. purpuratus* (e.g., north vs. south morphotypes previously described by Briones et al. (2012) [6] and those determined in our analyses).

To explore the morpho-genetic relationships, this study was exclusively conducted on male specimens of *P. purpuratus*, and the same individuals were used in both the sperm morphology and molecular analyses.

In *P. purpuratus*, previous studies reported double uniparental inheritance (DUI; [29,30]) of the mitochondrial genome [31,32]. Under the system of DUI, two types of mtDNA are transmitted to the offspring: The F type is derived from the egg, and the M type is derived from the sperm [33,34]. As in other mussels, both Vargas et al. (2015) [31] and Śmietanka et al. (2018) [32] observed in *P. purpuratus* that under DUI, females transmit their mtDNA (F type) to the offspring the same way as under SMI (i.e., strictly maternal inheritance). However, males have an additional mtDNA (M type) that is preferentially transmitted from fathers to sons. As a consequence, females are homoplasmic, but males are heteroplasmic, as they inherit mtDNA from both parents [35].

In mature male mytilids, the ratios of the F and M types in all tissues are variable (i.e., the testes contain predominantly the M genome, and somatic tissues predominantly contain the F genome) [36,37]. In *P. purpuratus*, sequences from 16S rRNA amplified with universal primers [38] showed the predominance of the M type and a lower occurrence of the F type in the somatic tissue of males (specifically in the mantle tissue) [31]. However, no information about the ratios of the F and M types in other somatic tissues, such as posterior adductor muscle, of this species exists. For this reason, to avoid possible problems associated with heteroplasmy, we performed a preliminary test for our 16S rRNA sequences from males. In this way, the presence of F- and/or M-type genomes in our mitochondrial sequences was determined in preliminary Bayesian inference analyses using a dataset of both male and female 16S rRNA sequences (the parameters used in this analysis are shown in Figure S2). 

### 2.6. DNA Extraction, Amplification, and Sequencing

The total genomic DNA of 1–10 males per location was used for molecular analyses (Table 1). DNA was extracted from small amounts of ethanol-preserved posterior adductor muscle tissue using the E.N.Z.A. TM Tissue DNA Kit (Omega Biotek, Inc., Norcross, Georgia).

For the study of intraspecific genealogies of *P. purpuratus* at the macro-scale level, partial mitochondrial 16S rRNA and nuclear 28S rRNA genes were amplified through Polymerase Chain Reaction (PCR) using the primers 16S-RA/16S-RB [38] and D23F/D6R [39], respectively. The PCR program included a denaturing cycle of 95 °C for 5 min, followed by 35 cycles of 95 °C for 45 s, an annealing temperature of 50 °C for the 16S marker and 52 °C for 28S marker for 45 s and 60 s, respectively, 72 °C for 60 s, and a final extension of 10 min at 72 °C. The PCR reaction had a total volume of 25 µL, containing between 10 and 20 ng of purified DNA, 2.5 µL of 5X NH4 Reaction Buffer (160 mM (NH_4_)_2_SO_4_, 670 mM Tris–HCl, pH 8.8) (1X of Green GoTaq^®^ Flexi Buffer), 10 mM of pre-mixed dNTPs, 2.5 mM of MgCl_2_, and 1.25 U of GoTaq Flexi DNA polymerase (Promega, Singapore). 

DNA extractions and PCR products were visualized with a UV transilluminator in 2% agarose gels. The purified amplifications were sequenced by Macrogen Inc. (Seoul, Republic of Korea) and STABVida (Lisboa, Portugal) with the reverse primers used in PCR amplifications. Sequencing was performed in capillary automatic sequencers (ABI 3730XL or ABI PRISM 3130, Applied Biosystems^®^, Foster, CA, USA).

The sequences were edited using ProSeq v 3.5 [40], and MSA was performed using the default parameters of the MAFFT online server platform [41], which used the multiple alignment based on the Fast Fourier Transform (FFT) [42]. All sequences were deposited in GenBank (accession numbers: OR196854–OR196919 for the 16S sequence and OR197218–OR197341 for the 28S sequence).

### 2.7. Bayesian Reconstructions and Genetic Structure

To infer some phylogenetic signals and to visualize the relationship between genetic divergence and sperm morphotypes inside the distribution of *P. purpuratus*, a Bayesian Inference (BI) was performed using the MCMC method for each genetic marker in MrBayes v. 3.2.7 [43]. In both the mitochondrial 16S rRNA and nuclear 28S rRNA datasets, two closely related Mytilidae species were used as outgroups: one *Brachidontes rodriguezii* (d’Orbigny 1842) male individual sampled for us in Las Grutas in the Neuquen province of Argentina (40°48′ S/65°05′ W) and one sequence downloaded from NCBI database for *Ischadium recurvum* (Rafinesque 1820). This last species was also used as an outgroup in studies of the *Brachidontes* complex, which included *P. purpuratus* populations [44,45,46]. For each gene dataset, the best-fitting substitution model determined with BIC in jModelTest v. 2.1.8 [47] was HKY. Each BI analysis included two independent runs, four chains, and a burn-in of 25% for a total of 10,000,000 generations, which were sampled every 1000 generations. The resulting consensus tree was then visualized and edited in FigTree v. 1.4.4 [48].

To explore the most likely number of gene pools (k) and their spatial boundaries, the population genetic structure of *P. purpuratus* was explored using the spatial Bayesian clustering models implemented in the GENELAND v. 4.9.2 R package [49]. For each gene dataset, a preliminary Bayesian exploration of the number of subpopulations (k) was performed in four genetic landscape scenarios: spatial/non-spatial combined with correlated and uncorrelated frequencies. The putative number of gene pools (k) was inferred from the modal value with the highest likelihood, and the spatial boundaries were detected as geographical areas with the lowest posterior probability of membership in clusters. For each landscape scenario, ten independent MCMC chains for k = 1–10 were assayed to estimate the most likely value of k while using 1,000,000 iterations and thinning of 100. Finally, an independent run was performed with the most likely k identified in the preliminary exploration using 5,000,000 iterations and thinning of 1000. 

To test the rationality of the 16S/28S clades and k-groups defined in the BI and GENELAND Bayesian clustering analyses, respectively, pairwise *F_ST_* values were computed, and hierarchical analyses of molecular variance (AMOVA) of the defined groups were executed in Arlequin v. 3.5 [50] using non-parametric permutation procedures with 10,000 iterations.

### 2.8. Estimation of Genetic Diversity and Demographic History

Standard genetic diversity indices, such as the number polymorphic sites (S), number of haplotypes (h), haplotype diversity (Hd), nucleotide diversity (Pi), and average number of nucleotide differences (k), were calculated using DnaSp v. 6 [51] for each adjusted acrosome morphotype and the previously identified lineages (i.e., clades and k-groups from the Bayesian analyses). 

To reveal the relationships among the haplotypes, a median-joining network [52] was constructed for each genetic dataset (16S and 28S) using the default parameters in the PopART software v 1.7 [53].

Finally, the demographic history of *P. purpuratus* was inferred for the genetic clades of the 16S dataset by means of the neutrality tests of Tajima’s D, Fu’s *Fs*, and Ramos-Onsins and Rozas’s R2, which were calculated using DnaSP v. 6 [51] with the Population Growth–Decline model. Pairwise mismatch distributions were calculated using DnaSP v 6 [51] to infer whether demographic expansions had occurred.

## 3. Results

### 3.1. Sperm Morphology Analyses

In the geographical distribution range analyzed in this study, sperm of *P. purpuratus* showed a primitive ect-aquasperm morphology [5,6], which is characteristic of free-spawning bivalves [16,17].

**Figure 2:**
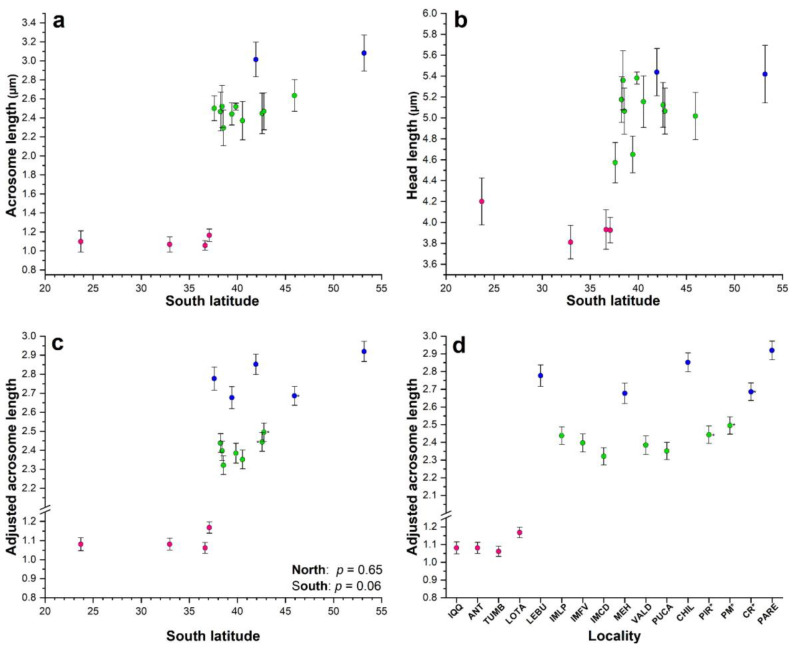
Scatterplot with error bars (±sd) for the relationship between mean acrosome length (**a**) and mean head length (**b**) according to latitude in *Perumytilus purpuratus*. The mean values for the adjusted acrosome length (±95% confidence interval) were graphed according to latitude (**c**) and locality (**d**). Colors represent the acrosome morphotypes—short (pink), intermediate (green), and long (blue)—as determined in the ANOVA (**a**,**b**) and ANCOVA (**c**,**d**). Localities’ codes are shown in Table 1.

Acrosomes and heads from a total of 480 spermatozoa were measured and statistically analyzed. The mean acrosome and head lengths varied geographically, showing the smallest sizes at northern localities up to Lota (37° S) and, thereafter, increasing steeply towards the southern localities (Table 2; Figure 2a,b). In fact, there were statistically significant differences among the localities (ANOVA for their rank-transformed lengths: *F*_15, 464_ = 112.19, *p* < 0.0001, for acrosomes; and *F*_15, 464_ = 131.37, *p* < 0.0001, for heads).

**Figure 3:**
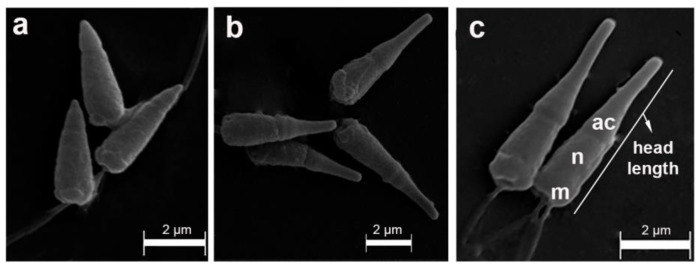
Scanning electron microscopy of sperm samples from *Perumytilus purpuratus* showing three different acrosome sizes, which represented three hypothetical morphotypes: short (**a**), intermediate (**b**), and long (**c**). **ac**: acrosome; **n**: nucleus; **m**: mitochondria. The maximum head length is shown.

In our study area, extensive acrosome and head variability was observed (Figure 3). The minimum and maximum values of the acrosome length and the acrosome/head ratio did not overlap between the 4 northern and the 12 southern localities (Figure S1), showing a clear distinction in acrosome size between them. In the northern localities, there were not any significant linear regressions among the mean values of the sperm statistics with respect to latitude (Table S1; *p* > 0.199), and in the southern localities, only the mean values of the acrosome length showed a positive and significant regression with respect to the latitude (Table S1; *p* = 0.010).

A short morphotype was observed in the northern localities up to Lota, where the shortest acrosome and head lengths—typical of the smaller sperm morphotype defined by Briones et al. (2012) [6] (Table 2; Figure 2a,b)—were significantly different from those of all other southern localities (adjusted Tukey test: *p* < 0.0001), except for the mean head length from Lebu, which did not significantly differ from that in Antofagasta (*p* = 0.115; Tables S2 and S3). However, there were not significant differences among them for the acrosome (adjusted Tukey test: *p* > 0.298; Table 3) and head lengths (adjusted Tukey test: *p* > 0.292), except for that in Antofagasta, which was significantly different from that in Valparaíso (*p* = 0.009; Table S3).

For the southern localities, Punta Arenas and Chiloé showed the longest mean acrosome and head lengths (Table 2, Figure 2a,b), which corresponded to the longest morphotype. This morphotype was significantly different from those in all the other localities (adjusted Tukey test: *p* < 0.0001; Tables S2 and S3), and there were no significant differences between them (adjusted Tukey test: *p* = 1.0; Tables S2 and S3). The other 11 southern localities showed intermediate sizes for acrosome and head lengths (Table 3, Figure 2a,b), representing the intermediate morphotype. 

For the ANCOVA, an outlier was removed to comply with the assumption of normality (K-S, *p* > 0.05). For the southern localities, reduced model 1 without the interaction term was the best candidate model. For the northern localities, the full model was the worst, and models 1, 2, and 3 were better and similar to Δ-AICc < 2 (Table S4), but we kept model 1 to permit appropriate comparisons with the southern localities. Accordingly, with this, the terms of the interaction between the covariable and locality (Head × Locality) were not significantly different for both northern localities (ANCOVA, *F*_3, 112_ = 1.55, *p* = 0.205) and southern localities (ANCOVA, *F*_11, 335_ = 0.68, *p* = 0.755). Therefore, the slopes were homogenous within each region: northern localities: Acrosome = 0.843 + 0.064 Head (*p* = 0.075; CL95% = −0.007–0.135); southern localities: Acrosome = 0.273 + 0.447 Head (*p* < 0.0001; CL95% = 0.374–0.520). The slopes between regions were significantly different, as their 95% confidence limits did not overlap. This implied a change in the shape in the sperm from the south with respect to those from the north, with an increase in the acrosome length at a higher rate that of the length of the head.

The adjusted acrosome length for the northern and southern localities did not vary significantly with latitude (linear regression, Adjusted acrosome length = 1.012 + 0.003 Latitude, *p* = 0.65, northern; Adjusted acrosome length = 1.440 + 0.027 Latitude, *p* = 0.055, southern; Figure 2c). 

For the northern localities, the adjusted acrosome did not significantly differ among Antofagasta, Valparaíso, and Tumbes (Tukey test: *p* > 0.786; Table 4 and Table S3; Figure 2d), but for Lota, the adjusted acrosome was longer and significantly different (Tukey test: *p* < 0.0001; Table 4 and Table S3; Figure 2d). All four northern localities showed a mean adjusted acrosome of 1.098 µm (CL = 1.090–1.106), which corresponded to the short morphotype.

For the southern localities, the adjusted acrosome length was segregated into two different groups that differed statistically (Tukey test: *p* < 0.05, as their confidence limits overlapped) (Table 4; Figure 2d). A group comprising seven localities—three from Isla Mocha (IM) (Punta Los Piures, Faro Viejo, and Caleta Derrumbre) and four from the continental territory (Valdivia, Pucatrihue, Punta Pirámide, and Puerto Madryn)—with intermediate acrosomes showing a mean adjusted acrosome of 2.441 µm (CL = 2.423–2.460), which corresponded to the intermediate morphotype. The other group consisted of five localities—Lebu, Mehuín, Chiloé, Comodoro Rivadavia, and Punta Arenas—with a longer mean adjusted acrosome of 2.729 µm (CL = 2.683–2.774), which corresponded to the long morphotype.

### 3.2. Molecular Analyses

A total of one hundred ninety sequences from male *P. purpuratus* specimens collected at 21 different localities (refer to Table 1) were included in the following molecular analyses. This dataset encompassed approximately 74% of the species’ documented distributional ranges [13,14]. For the 16S and 28S markers, an average of three and six individuals per location, respectively, were sequenced.

After alignment and trimming the ends, 66 and 124 sequences of 406 and 660 bp fragments were obtained for the mitochondrial rRNA 16S and the nuclear rRNA 28S genes, respectively.

The preliminary BI analysis performed on the mitochondrial 16S sequences from males and females of *P. purpuratus* confirmed the double uniparental inheritance (DUI) that was previously reported for this species [31,32]. In consequence, the genetic divergence displayed a robust separation between the F- and M-types (Figure S1). Using posterior adductor muscle as a somatic tissue, our results showed that the F-type mitochondrial genome was present only in females, and the M-type was observed only in males; this is the first evidence of the presence of the M-type genome in the posterior adductor muscle of males of *P. purpuratus* (Figure S2). The absence of the F-type genome in the posterior adductor muscle of males allowed us to discard the assumption of the presence of both F- and M-type genomes in this kind of tissue. Therefore, and due to the relationship between the M-type genome and sperm, since it is transmitted to the offspring by sperm [33,34], in this work, we opted to explore the molecular divergence of the M-type genome of *P. purpuratus*.

### 3.3. Bayesian Reconstructions, Genetic Structure, and the Relationship with Acrosome Morphotypes

**Figure 4:**
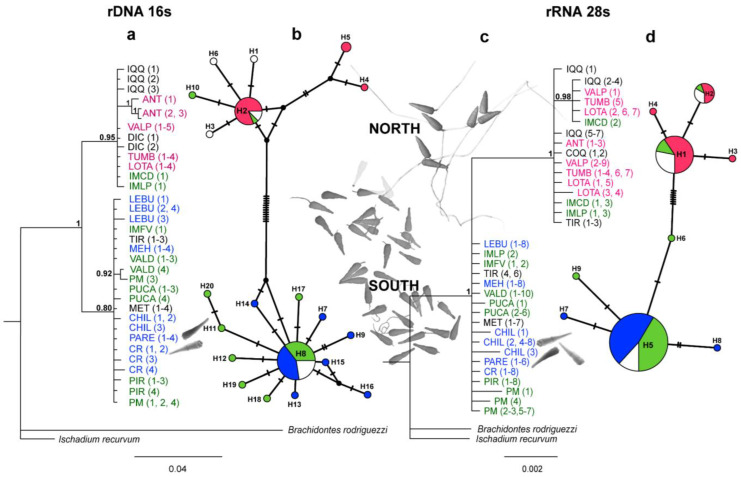
Phylogenetic relationships and haplotype network based on the rRNA 16S (M-type) and rRNA 28S genes for *Perumytilus purpuratus*. (**a**,**c**): Phylogenetic tree from Bayesian inference analysis with Bayesian posterior probabilities values near the branches. Numbers in parentheses following the species name indicate the number of sequences (see Table 1). (**b**,**d**): Median-joining network with node sizes proportional to the frequencies of haplotypes and mutational steps symbolized by dashes. Colors represent the localities with the acrosome sperm morphotypes—short (pink), intermediate (green), and long (blue)—estimated via ANCOVA. The names are in black, and white haplotypes indicate localities without sperm morphological information. The localities’ codes are shown in Table 1.

The trees of the Bayesian analyses based on the 16S and 28S genes showed two well-supported clades (posterior probabilities values >95%) that were sisters to the *Brachidontes rodriguezii* and *Ischadium recurvum* outgroups (Figure 4). In both the 16S and 28S sequences, the phylogenetic separations were similar and were related to the acrosome morphotypes (i.e., north vs. south clades; Figure 4). On the one hand, in the 16S/28S analyses, the north clade was formed by individuals from the northern localities (Iquique to Lota, i.e., all localities with a short morphotype) and by three localities from 38° S (i.e., IM Caleta Derrumbe and IM Punta Los Piures, with an intermediate morphotype for 16S/28S trees, plus three sequences from Tirúa in the 28S tree; see Figure 4). On the other hand, the south clade showed strong support, with the rest of the localities showing both intermediate and long morphotypes (Figure 4). Even though the results of the Bayesian inference were similar between the 16S and 28S datasets, the tree topologies showed some differences. While the 16S sequences showed two independent clades (north vs. south), in the 28S analysis, the north clade was nested within the basal south clade (Figure 4).

**Figure 5:**
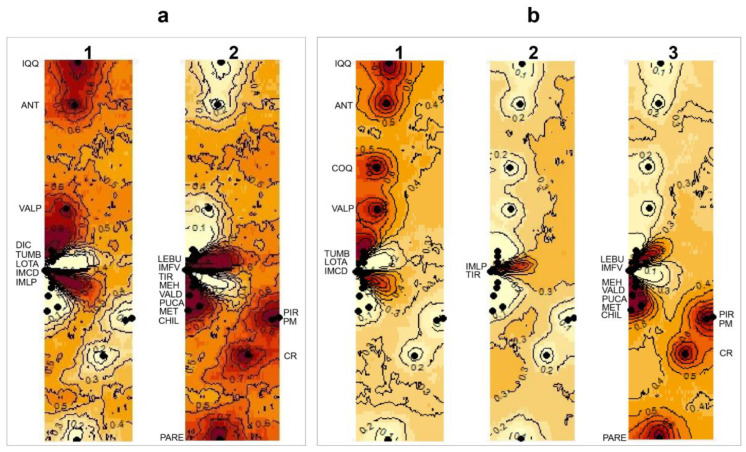
Maps of the isoclines of the posterior probabilities belonging to the different clusters inferred from GENELAND for *Perumytilus purpuratus*: (**a**) mitochondrial (16S) and (**b**) nuclear (28S) sequences using the spatial model with correlated allele frequencies. Black dots correspond to the localities. The inferred clusters are shown by a number on the map. For (**a**), 1 and 2 correspond to the north and south clades, respectively (i.e., k = 2); and for (**b**), 1, 2 and 3 correspond to the north, intermediated and south clades, respectively (i.e., k = 3). The darker and lighter shadings are proportional to the posterior probabilities of membership in the clusters, with darker (red) areas showing the highest probabilities of clusters. The localities’ codes are shown in Table 1.

After exploring the four landscape scenarios of the Bayesian clustering analysis implemented in GENELAND, the spatial model combined with the correlated frequency was selected for both the 16S and 28S datasets. The correlated frequency model has been described as a more biologically grounded way to make inferences, and it has been observed that using the correlated frequency model could be more powerful when detecting subtle admixture differentiation [49]. Between the 16S and 28S sequences, the results of the Bayesian clustering analyses showed different k-groups. In the 16S dataset, the two main clusters observed (north vs. south clusters, K = 2) were composed of the same sequences as those from the BI analysis (Figure 5a). However, in the 28S dataset, three main clusters were observed (K = 3): (I) the north cluster with the localities from Iquique to Lota (20° S–37° S) plus IM Caleta Derrumbe (38° S; Figure 5(b1)); (II) a transition cluster composed of IM Punta Los Piures and Tirúa (38° S; Figure 5(b2)); (III) the south cluster with the rest of the southern localities (Figure 5(b3)).

**Figure 6:**
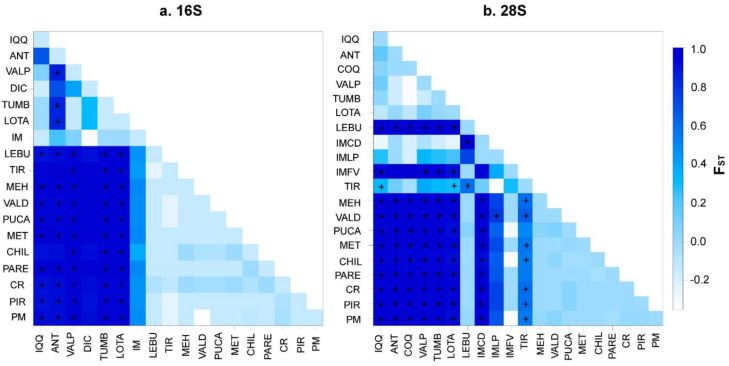
Matrix of pairwise F_ST_ values computed with Arlequin v. 3.5 to illustrate the population connectivity of *Perumytilus purpuratus* for all population comparisons from the (**a**) 16S and (**b**) 28S sequences. The + symbol indicates the F_ST_ *p*-values that were significantly different from zero (*p* < 0.05). The color grading reflects the degree of divergence and corresponds to the F_ST_ values indicated in the legend. The localities’ codes are shown in Table 1.

Despite these differences, both Bayesian methods suggested divergent lineages (clades) inside the distribution of *P. purpuratus* and a limited gene flow between these geographical regions (i.e., north, transition, and south). In fact, the panmixia hypothesis was rejected in the hierarchical AMOVA (*p* < 0.05; Table 5), suggesting a genetic structure inside the geographical distribution of *P. purpuratus*. The AMOVA results were consistent when using the geographical partitions observed in both the BI and GENELAND analyses. For both the mitochondrial (16S) and nuclear (28S) markers, the percentages of variation were higher among the groups (>94%) and lower among the samples within the groups (<1.94%) and within the samples (<6.6%; see Table 5). Accordingly, the pairwise *F_ST_* values were significantly different between the compared groups (Figure 6).

### 3.4. Genetic Diversity

Among the 406 and 660 bp of the alignment lengths of the 16S and 28S sequences, respectively, 33 (8.1%) and 15 (2.3%) were polymorphic sites; of those, 14 and 6 were singleton variable sites (3.5% and 0.9%, respectively), and 19 and 9 were parsimony informative sites (4.7% and 1.4%, respectively; Table 6). Of the total of the sequenced individuals, the number of haplotypes (h), haplotype diversity (Hd), nucleotide diversity (Pi), and nucleotide differences (k) were greater for the 16S rRNA dataset than for the 28S rRNA dataset (Table 6). For the 16S marker, the indices estimated with the adjusted acrosome revealed a high genetic diversity for the intermediate morphotype (Table 6a). However, the indices estimated with the BI analyses showed higher values for the north clade (16S) and for the transition cluster (28S) (Table 6b,c).

Of the haplotypes identified for the 16S and 28S markers (h = 20 and 9, respectively), only three were shared haplotypes. The haplotype networks showed a non-random association, which was related to the acrosome morphotypes, genetic clades, and, therefore, their geographic origin (i.e., north vs. south zones, Figure 4). Thus, the localities with the short morphotype showed haplotypes that were exclusively related to the north clade (northern haplotypes: 16S h = 2, 4, 5 and 28S: h = 1–2, Figure 4b,d); of the localities with the intermediate and long morphotypes, all (with the exception of IM Caleta Derrumbe, IM Punta Los Piures, and Tirúa—see Figure 4b,d) showed haplotypes from the south clade. In accordance with the previous Bayesian results (Figure 4), IM Caleta Derrumbe and IM Punta Los Piures (38° S) showed the northern haplotypes in the 16S network (Figure 4b). In the 28S analysis, all sequences from IM Caleta Derrumbe showed northern haplotypes, but IM Punta Los Piures and Tirúa showed both northern and southern haplotypes (Figure 4d). Finally, in addition to the shared haplotype from the south clade, two private haplotypes were observed in Chiloé (Figure 4d).

### 3.5. Demographic History

**Figure 7:**
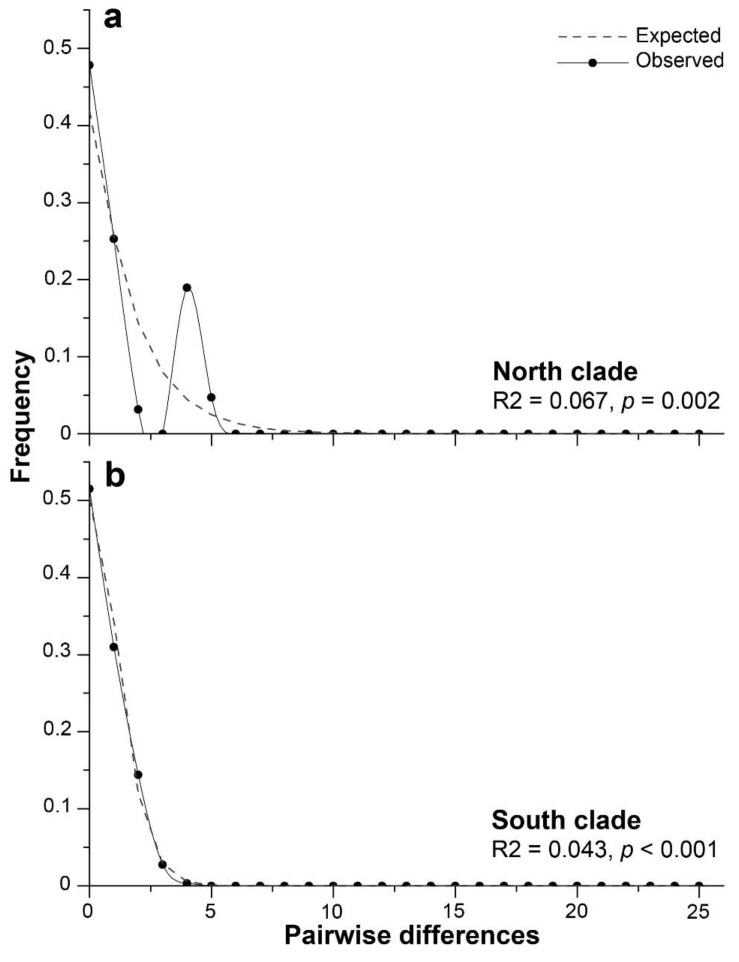
Bimodal (**a**) and unimodal (**b**) patterns from the mismatch distribution analysis for the 16S gene M-type of *Perumytilus purpuratus* showing the expected and observed pairwise differences between the sequences of the north clade (**a**) and the south clade (**b**). The results of Ramos-Onsins and Rozas’s test (R2) are shown.

The neutrality tests performed for the 16S genetic clades suggested a recent population expansion (Table 7). For the south clade, all neutrality tests were statistically significant (*p* < 0.01; Table 7), but for the north clade, only the R2 test of Ramos-Onsins and Rozas was statistically significant (*p* < 0.01; Table 7). When all samples were regarded as a whole, Tajima’s *D*, Fu’s *Fs*, and the R2 statistic values were statistically not significant (*p* > 0.10). The pairwise mismatch distribution used to visualize evidence of the population expansion suggested by the previous neutrality tests showed a bimodal distribution for the north clade, which was consistent with allopatric divergence, followed by population growth (Figure 7a). For the south clade, a unimodal mismatch distribution represented the expanding populations (Figure 7b).

## 4. Discussion

Along a latitudinal gradient of ca. 7180 km of coastline—from the Pacific to the Atlantic Ocean, we showed evidence of a discontinuity in genetic and spermatic morphotypes at 37° S, with two distinct genetic lineages (clades) in males of *P. purpuratus*. Although the presence of two lineages could be indicative of cryptic species for this intertidal mussel [6,21,22], we interpreted our observation of a hybridization zone at 38° S as a strong signal of incipient or *in progress* (*in fraganti*) speciation processes occurring in *P. purpuratus* [6].

### 4.1. Sperm Morphology

In *Perumytilus purpuratus*, there was evidence of geographical variability in sperm sizes (for the mean acrosome and head lengths). The AICc-selected model 2, an ANCOVA with Locality and Head as predictor variables of Acrosome length, was better than the others, including model 3 (ANOVA), showing that the north lineage was characterized by the shorter morphotype and the south lineage was characterized by localities with two different intermediate and long morphotypes, as shown in Figure 2c,d.

Thus, our morphological findings revealed the following: (1) three sperm morphotypes along the Chilean and Argentinean distributional range of *P. purpuratus*; (2) a main morphological break between northern and southern localities situated in the Pacific Ocean at 37° S; (3) along with an intermediate morphotype, and a novel morphotype, which was longer than those that were previously reported [5,6,54] and was observed in southern localities of both the Pacific and Atlantic.

Several researchers have suggested that sperm traits are species-specific and, therefore, are valuable for studying taxonomic affinities among species (e.g., [5,6,7,55,56]). In this same line, this detailed morpho-anatomical, ultrastructural, and molecular work in bivalves highlights that the ultrastructural characteristics of sperm are among the best morphological features for determining bivalve clades [57]. Therefore, a relationship between sperm morphotypes and genetic structure was expected.

### 4.2. Linking Sperm Morphotypes with Genetic Divergence

In agreement with previous reports [31,32], we confirmed DUI for the mitochondrial genome of *P. purpuratus*. In our preliminary assay, the 16S sequences from males showed a unique mitochondrial genome (M type) in the posterior adductor muscle, since no male sequences were observed in the female clade (see Figure S2).

In molecular analyses of mussels, DNA is generally extracted from the posterior adductor muscle (or mantle) tissue. In *Brachidontes* genera (a closely–related species), DUI has been found in some species, but not in others [58,59], and some researchers have opted for isolating DNA from the posterior adductor muscle to avoid using male gonadal tissue, which is enriched with paternally transmitted ‘‘male’’ mitochondrial genomes (i.e., M type) [44,45]. This was in accordance with our preliminary assay, since, in the female and male sequences of our outgroup species *Brachidontes rodriguezii*, no gender-associated divergence was observed (see Figure S2). Nevertheless, according to Trovant et al. (2013) [45], the infiltration of the muscle tissue by the male germ line is unlikely; therefore, it is expected to be dominated by maternally transmitted mitochondria (F type), irrespective of the gender of the individual mussel sampled [45]. However, the opposite was observed in males of *P. purpuratus*, indicating that this should not be considered a rule within the Mytilidae family.

The molecular results obtained using mitochondrial 16S M-type and nuclear (28S) sequences showed genetic divergence and the presence of a phylogeographic structure along the distributional range of *P. purpuratus*, with two well-differentiated north and south clades, which we have now designated as the north lineage and the south lineage. These results were congruent with the divergence observed in the 16S F type (i.e., according to our preliminary assay; Figure S2) and with previous reports using mitochondrial (16S, COI) and nuclear (18S, 28S) molecular markers [6,21,22,46]. Our results also agreed with the regional genetic differentiation between *P. purpuratus* from the southeastern Pacific (Punta de Tralca, 33°26′ S) and southwestern Atlantic (Puerto Lobos, 41°42′ S) found when using microsatellite markers [60]. Overall, these findings are evidence for restricted gene flow between the north and south lineages.

In sessile marine broadcast spawners, such as *P. purpuratus*, the connectivity level and genetic flow between distant populations can depend on multiple factors—for example, the larval dispersal capacity, contemporary oceanography, and historical climatic events (see [22]). However, the relationship between genetic divergence and the acrosome morphotypes reported here, with the short morphotype associated with the north lineage and both intermediate and long morphotypes associated to the south lineage, suggests that reproductive barriers and, therefore, reproductive isolation could be the current mechanism preventing gene flow between lineages.

If we consider the species specificity of spermatozoan traits to differentiate closely related species (e.g., acrosome size as a species-conservative trait), then each morphotype must be a different species. However, our morpho-genetic results were contrasting because the two genetic lineages were associated with three sperm morphotypes.

On the one hand, the presence of two genetic lineages (the present work and [6,21,22,46]) suggests the evolutionary scenario of cryptic speciation [6,22,54], which means two species with prezygotic isolation, i.e., barriers that prevent fertilization, and, therefore, genetic exchange between previously interbreeding populations [61,62]. However, the lack of genetic differentiation observed between the intermediate and long morphotypes—as both were grouped within the south lineage—may also suggest an evolutionary scenario of incipient species within the southern region. In reproductive terms, this scenario likely involves postzygotic isolation, which is characterized by barriers occurring after zygote formation, leading to the production of non-viable or infertile offspring [61,62].

### 4.3. Cryptic and Incipient Species Scenarios: An Evolutionary Perspective

According to our results, the scenarios of both cryptic and incipient species are probable. In this way, we hypothesize that the short morphotype represents a cryptic species and that individuals with intermediate and/or long morphotypes could correspond to incipient species.

In an evolutionary context, the association between genetic divergence and adjusted acrosome morphotypes that we observed could be explained by historical events, such as the LGM [6,21,22], where glacial sheets most likely disrupted larval dispersal and, consequently, the genetic connectivity between the southern and northern populations. For example, glaciation–deglaciation events have shaped the geomorphological configuration of the Chilean coast. In this context, the coastline between Perú and Canal de Chacao (north of Chiloé island, ca. 41°47′ S) is continuous, smooth, and without breaks or major geographical features [63]. However, from Chiloé to Cabo de Hornos (~56° S), the area known as the Chilean archipelago, the coastal geomorphology is complex and characterized by a profusion of gulfs, fjords, and channels resulting from the combined effects of tectonic processes and glaciation [63] (Figure 1). During the Last Glacial Maximum (LGM), which is dated 23–25 ka ago for Patagonia [64,65], icefields covered all of southern Chile, from the Chilean Lake District (40° S) to Bahía Inútil in Tierra del Fuego (53.5° S) [64], creating a great geographical barrier or breaks in habitat continuity between the southern and northern populations of *P. purpuratus*.

In hypothetical terms, evidence has indicated that the north lineage corresponds to isolated populations that remained unaffected by icefields during the LGM period, and, therefore, larval connectivity and gene flow between the populations remained. This could explain the lower morphological variability in sperm observed for the north lineage, where the short morphotype remained relatively constant along ca. 1744 km of coastline (from Antofagasta to Lota; Figure 2c). In the Antofagasta area (23° S), fossil records of *P. purpuratus* observed in molluscan assemblages of the last interglacial period (early Pleistocene ca. 120 ka [53,54,66,67]) support this hypothesis. Consequently, our demographic analyses using mtDNA M-type showed a bimodal mismatch distribution for the north lineage (Figure 7a). Dawson et al. (2002) [68] suggested that a bimodal mismatch distribution is attributable to a historically differentiated allopatric population. In addition, bimodal shape may be sensitive to the age of the expansion, with the right peak representing an older expansion and the other peak a recent expansion [69,70]. In this way, the north lineage of *P. purpuratus* might have a more complex evolutionary history than that of the south lineage. Instead, the mismatch distribution for the south lineage was unimodal in shape and closely fitted the expected distribution under the sudden expansion model (Figure 7b), that may be attributable to a more recent population expansion than that of the north lineage. This finding was consistent with those of analyses conducted by Trovant et al. (2015) [21] when using the COI molecular marker, as they (assuming a mutation rate of 0.19 substitutions/Myr) estimated different population expansion timings for each *P. purpuratus* lineage, with a northern expansion that could have started around 15 ka (end of the Pleistocene) and a more recent expansion for the south lineage, where the largest change in population size could have occurred during the Holocene (11.5 to 3.5 ka BP). This could explain the variability in sperm morphotypes assigned to the south lineage, where the two morphotypes were distributed along ca. 5436 km of coastline, without a significative relationship with latitude (Figure 2c).

Furthermore, our comprehensive sampling effort enabled us to precisely determine the latitudinal position of the morpho-genetic break, which was identified at 37° S on the Pacific coast. This location was notably farther north than the area covered during the Last Glacial Maximum (LGM). This evidence and the observation of the long morphotype in the break zone (Lebu) strongly suggest the postglacial recolonization of the south lineage of *P. purpuratus* and support the hypothesis of ice-free refugia or suitable niches within these quaternary glacial areas [71]. Therefore, we hypothesized that on the Pacific coast, the postglacial recolonization of the south lineage of *P. purpuratus* reached 37° S and that the different sperm morphotypes of the south lineage could have originated in a distinct glacial refugium during the LGM period.

In other marine species inhabiting southern Chile, evidence of historical influences (i.e., the LGM) on cladogenesis also have been reported, such as in macroalgae [72,73,74], fish [75], arthropods [76], and gastropods [71,77]. For example, in the red algae *Mazzaella laminarioides*, Montecinos et al. (2012) [72] also localized a genetic break at 37° S that, according to these authors, could have originated due to transient habitat discontinuities driven by episodic tectonic uplifting of the shoreline around the Arauco region (37°–38° S). In addition, three divergent lineages—northern (28°55′ S to 32°37′ S), central (34°05′ S and 37°38′ S), and southern (39°40′ S to 54°03′ S)—and evidence of postglacial recolonization from a northern glacial refugium area were observed in this species [72]. In kelp, genetic disjunction between Patagonian (49°–56° S) and northern populations (32°–44°) was observed in *Durvillaea antarctica* [73]; in *Macrocystis pyrifera*, Macaya and Zuccarello (2010) [74] reported a genetic break at 42° S (Chiloé Island) and shared haplotypes among some of the subantarctic islands and southern-central Chile, suggesting a recent colonization of the subantarctic region. In *P. purpuratus*, we observed shared haplotypes at the intra-lineage level, which was a signal of gene flow disruption between the north and south lineage. However, an exception was observed for IM Caleta Derrumbe, IM Punta Los Piures, and Tirúa (localities situated at 38° S; see Figure 1), where some individuals showed the typical southern haplotypes, but others showed northern haplotypes with both mitochondrial (16S) and nuclear (28S) molecular markers. As a result, these individuals were positioned inside both the north and south lineage (see Figure 4). We interpreted this outcome as evidence of a local hybridization zone at 38° S, suggesting the presence of an incipient speciation process within the south lineage.

### 4.4. Incipient Species and Postzygotic Isolation inside the South Lineage of P. purpuratus?

Our study allowed us to determine a possible hybridization zone at 38° S in the Pacific near the morpho-genetic boundary situated at 37° S. At 38° S, two principal localities were sampled: Tirúa (38°20′ S) in the continental territory and Isla Mocha (Punta Los Piures, Caleta Derrumbe, and Faro Viejo), situated 35 km in front of Tirúa in the insular territory. With exception of IM Faro Viejo, the individuals from these localities showed haplotypes that were typical of both the north and south lineage (see Figure 4 and Figure 5). It should be noted that we did not have sperm morphological information for Tirúa, but according to our findings, we hypothesized that the intermediate morphotype should be present in this locality. In this way, individuals at these localities showed the intermediate sperm morphotype, which was the morphotype attributed to the south lineage. However, we consider this finding as the first evidence of a secondary contact zone in *P. purpuratus*, with individuals showing interspecific hybridization, where northern alleles probably introgressed into the gene pool of the south lineage.

In theory, a secondary contact zone is formed when two populations or lineages that have diverged due to genetic drift or selection during a period of geographic isolation come into contact [78,79,80]. As was discussed previously, this finding was consistent with the hypothetical origin of divergence between the *P. purpuratus* lineages and the later demographic expansion of the south lineage until 37° S. In this way, when secondary contact zones are established, genetic isolation can be maintained by prezygotic and/or postzygotic mechanisms [80].

In the case of postzygotic isolation, the mechanisms for reproductive isolation have not been completed; therefore, hybrid offspring are produced through introgressive hybridization processes and maintained through intrinsic genetic incompatibilities or extrinsic causes of selection against hybrids [80]. According to introgressive hybridization, the movement of alleles from one species into the gene pool of another divergent species occurs by means of repeated backcrossing of an interspecific hybrid with one of its parent species [81,82]. In mussels, hybridization zones have been well studied, especially in the *Mytilus* species complex, which hybridizes naturally [83,84,85,86,87], and the general expectation is that hybridization could lead to offspring with reduced fitness—for example, sterility or inferior viability—which is probably due to the assumption that crosses between divergent genotypes will always disrupt co-adapted genomes; however, fitness for hybrids can range to the highest to the lowest (see [88]). In fact, several studies of *Mytilus* have shown reduced hybrid fitness, such as through larval inviability and an early heterosis rate [89], abnormal larvae [90,91], and high levels of sterility [92]. In our work, the lowest frequency of “hybrid” specimens could be a signal of reduced fitness.

Regarding this, the genetical analyses performed using the GENELAND package, which is suitable for hybrid zone inference [93], showed a transition cluster in IM Punta Los Piures and Tirúa (Figure 5b), i.e., the localities in which individuals with both northern and southern haplotypes were observed. Nevertheless, the molecular markers used in this study (16S and 28S) are not suitable for the detection of hybrids. Therefore, whether individuals from these localities correspond to hybrids must be determined using specific markers for introgression and hybridization detection, such as SNPs, and this marker should be used to evaluate our hypothesis of a hybridization zone at 38° S.

## 5. Conclusions

Along a latitudinal gradient of ca. 7180 km of coastline—from the Pacific to the Atlantic Ocean—our work showed correlations between the intraspecific variations in spermatozoan traits and the genetic structure of males of *P. purpuratus*. Additionally, the evidence of genetic admixture between lineages (i.e., in Isla Mocha–Tirúa) and a probable hybridization zone at this latitude (38° S) strongly suggested that *P. purpuratus* is under incomplete reproductive isolation, where the presence of mechanisms of reproductive isolation could be manifested between lineages of *P. purpuratus* and, therefore, could be in an incipient speciation process. In conclusion, our findings contribute to a better understanding of the evolutionary history of *P. purpuratus*; nevertheless, more experimental studies are needed to confirm the occurrence of incipient speciation and a hybridization zone for this species, including a careful assessment of the morpho-genetic variation by means of additional analyses, such as those of laboratory crosses at intra/interspecific level and the study of reproduction-related proteins. From an evolutionary perspective, these are valuable because of their contribution to reproductive isolation between species and for the formation of new species [94,95,96,97]. In addition, our work contributes to the knowledge on paternally inherited mtDNA divergence at the geographic macroscale in *P. purpuratus*. Nevertheless, forthcoming research should further investigate the evolutionary significance of the male mitotype.

Finally, we consider that despite uncertainties in the taxonomy of *P. purpuratus*, the morpho-genetic divergence between the north and south lineages of *P. purpuratus* indicates that they do not constitute the same Evolutionary Significant Unit and, therefore, should be considered as separate entities.

## Figures and Tables

**Table 1:** The sampling locality, code, coordinates, country/ocean, number of sequences (seqs) for the 16S and 28S markers, and sperm data of *P. purpuratus* sampled in intertidal zones in 21 localities. For the 16S and 28S molecular markers, averages of three and six individuals per locality, respectively, were used. The sperm data corresponded to measurements of spermatozoa for an average of three individuals per locality. An asterisk indicates that the sperm data came from Briones et al. (2012) [6].

Sampling Location	Code	Coordinates	Country/Ocean	16S seqs	28S seqs	Sperm Data
1. Iquique	IQQ	20°14′ S/70°09′ W	Chile/Pacific	3	7	No data
2. Antofagasta	ANT	23°42′ S/70°26′ W	Chile/Pacific	3	3	30 *
3. Punta Choros, Coquimbo	COQ	29°14′ S/71°27′ W	Chile/Pacific	No data	2	No data
4. Valparaíso	VALP	32°57′ S/71°33′ W	Chile/Pacific	5	9	30 *
5. Dichato, Tomé	DIC	36°32′ S/72°56′ W	Chile/Pacific	2	No data	No data
6. Tumbes	TUMB	36°38′ S/73°05′ W	Chile/Pacific	4	7	30
7. Lota	LOTA	37°04′ S/73°09′ W	Chile/Pacific	4	7	30
8. Lebu	LEBU	37°35′ S/73°05′ W	Chile/Pacific	4	8	30
9. Tirúa	TIR	38°20′ S/73°30′ W	Chile/Pacific	3	5	No data
10. Isla Mocha Punta Los Piures	IMLP	38°21′ S/73°52′ W	Chile/Pacific	1	3	30
11. Isla Mocha Faro Viejo	IMFV	38°22′ S/73°56′ W	Chile/Pacific	1	3	30
12. Isla Mocha Caleta Derrumbe	IMCD	38°24′ S/73°54′ W	Chile/Pacific	1	2	30
13. Mehuín	MEH	39°25′ S/73°13′ W	Chile/Pacific	4	8	30
14. Valdivia	VALD	39°50′ S/73°23′ W	Chile/Pacific	4	10	30 *
15. Pucatrihue	PUCA	40°32′ S/73°42′ W	Chile/Pacific	4	6	30
16. Metri	MET	41°35′ S/72°42′ W	Chile/Pacific	4	7	No data
17. Chiloé	CHIL	41°55′ S/74°02′ W	Chile/Pacific	3	8	30
18. Punta Arenas	PARE	53°10′ S/70°55′ W	Chile/Pacific	4	6	30
19. Comodoro Rivadavia	CR	45°56′ S/67°33′ W	Argentina/Atlantic	4	8	30
20. Punta Pirámide	PIR	42°34′ S/64°17′ W	Argentina/Atlantic	4	8	30
21. Puerto Madryn	PM	42°45′ S/65°01′ W	Argentina/Atlantic	4	7	30
Total dataset				66	124	480

**Table 2:** Descriptive statistics of the morphometric data of the sperm parameters: acrosome length (µm), head length (µm), and the acrosome/head length ratio (%) for *Perumytilus purpuratus* from sixteen localities. Mean ± standard deviation (SD), minimum (Min), and maximum (Max) values are shown.

Location	N	Acrosome Length (µm)			Head Length (µm)			Acrosome/Head Ratio (%)		
		Mean ± (SD)	Min	Max	Mean ± (SD)	Min	Max	Mean ± (SD)	Min	Max
Antofagasta	30	1.099 ± 0.111	0.807	1.336	4.201 ± 0.226	3.834	4.663	26.245 ± 2.996	19.376	31.828
Valparaiso	30	1.069 ± 0.081	0.888	1.194	3.811 ± 0.160	3.447	4.079	28.075 ± 2.268	24.409	34.046
Tumbes	30	1.059 ± 0.050	0.976	1.162	3.931 ± 0.189	3.578	4.293	26.967 ± 1.532	24.576	30.684
Lota	30	1.165 ± 0.065	1.008	1.291	3.926 ± 0.121	3.709	4.267	29.669 ± 1.389	26.988	32.210
Lebu	30	2.502 ± 0.131	2.296	2.780	4.572 ± 0.193	4.172	4.914	54.722 ± 1.741	51.159	57.590
IM Punta Los Piures	30	2.468 ± 0.204	1.968	2.858	5.176 ± 0.218	4.913	5.749	47.662 ± 3.142	39.452	54.540
IM Faro Viejo	30	2.520 ± 0.222	2.009	2.917	5.360 ± 0.283	4.914	5.891	47.03 ± 3.452	35.589	52.026
IM Caleta Derrumbe	30	2.295 ± 0.189	1.968	2.703	5.065 ± 0.221	4.653	5.574	45.318 ± 3.192	39.449	50.829
Mehuín	30	2.442 ± 0.116	2.280	2.811	4.651 ± 0.176	4.344	5.035	52.497 ± 1.350	50.309	55.829
Valdivia	30	2.519 ± 0.036	2.451	2.579	5.382 ± 0.058	5.240	5.494	46.809 ± 0.973	45.307	48.826
Pucatrihue	30	2.371 ± 0.201	1.940	2.697	5.155 ± 0.247	4.442	5.628	45.982 ± 3.052	39.327	51.796
Chiloé	30	3.014 ± 0.182	2.696	3.353	5.438 ± 0.226	5.023	5.881	55.42 ± 2.019	51.725	59.472
Punta Arenas	30	3.082 ± 0.190	2.831	3.686	5.419 ± 0.276	4.889	6.058	56.882 ± 2.125	53.598	62.311
Comodoro Rivadavia	30	2.636 ± 0.167	2.374	3.067	5.018 ± 0.225	4.649	5.403	52.535 ± 2.180	47.622	57.661
Punta Pirámide	30	2.447 ± 0.213	1.919	2.831	5.124 ± 0.214	4.870	5.627	47.772 ± 3.711	35.583	55.366
Puerto Madryn	30	2.469 ± 0.193	2.053	2.930	5.065 ± 0.220	4.636	5.474	48.735 ± 2.904	38.831	54.920

**Table 3:** Matrix of the adjusted *p*-values (upper diagonal) obtained through pairwise comparisons between localities in the ANOVA post hoc Tukey test. Non-significant differences (*p* > 0.05) are shown in bold. Below the diagonal, the pairwise comparisons with non-significant differences (*p* > 0.05 shown in the bold upper diagonal) are represented by degraded colors: pink = short, green = intermediate, and blue = long morphotype. Dark colors indicate that *p*-value = 1, and light gray indicates significant differences (*p* < 0.05). The localities’ codes are shown in Table 1.

Locality	ANT	VALP	TUMB	LOTA	LEBU	IMLP	IMFV	IMCD	MEH	VALD	PUCA	CHIL	PIR	PM	CR	PARE
**ANT**	—	**1.000**	**0.999**	**0.967**	0.000	0.000	0.000	0.000	0.000	0.000	0.000	0.000	0.000	0.000	0.000	0.000
**VALP**		—	**1.000**	**0.638**	0.000	0.000	0.000	0.000	0.000	0.000	0.000	0.000	0.000	0.000	0.000	0.000
**TUMB**			—	**0.298**	0.000	0.000	0.000	0.000	0.000	0.000	0.000	0.000	0.000	0.000	0.000	0.000
**LOTA**				—	0.000	0.000	0.000	0.000	0.000	0.000	0.000	0.000	0.000	0.000	0.000	0.000
**LEBU**					—	**1.000**	**1.000**	0.000	**0.681**	**1.000**	**0.162**	0.000	**0.982**	**1.000**	0.033	0.000
**IMLP**						—	**0.975**	0.004	**0.998**	**0.851**	**0.814**	0.000	**1.000**	**1.000**	0.001	0.000
**IMFV**							—	0.000	**0.289**	**1.000**	0.033	0.000	**0.797**	**0.990**	**0.163**	0.000
**IMCD**								—	**0.188**	0.000	**0.725**	0.000	0.022	0.002	0.000	0.000
**MEH**									—	**0.114**	**1.000**	0.000	**1.000**	**0.993**	0.000	0.000
**VALD**										—	0.008	0.000	**0.519**	**0.914**	**0.379**	0.000
**PUCA**											—	0.000	**0.979**	**0.722**	0.000	0.000
**CHIL**												—	0.000	0.000	0.000	**1.000**
**PIR**													—	**1.000**	0.000	0.000
**PM**														—	0.001	0.000
**CR**															—	0.000
**PARE**																—

**Table 4:** Two independent matrices of the adjusted *p*-values (upper diagonal) obtained through pairwise comparisons between localities in the ANCOVA post hoc Tukey test are shown: **A.** for northern localities from ANT to LOTA; **B.** for southern localities from LEBU to PARE. Non-significant differences (*p* > 0.05) are shown in bold. Below the diagonal, the pairwise comparisons with non-significant differences are represented by degraded colors: pink = short, green = intermediate, and blue = long morphotype. Dark colors indicate that *p*-value = 1, and light gray indicates significant differences (*p* < 0.05). Localities’ codes are shown in Table 1.

**A**	**Locality**	**ANT**	**VALP**	**TUMB**	**LOTA**								
	**ANT**	—	1.000	0.829	0.002								
	**VALP**		—	0.786	0.000								
	**TUMB**			—	0.000								
	**LOTA**												
**B**		**LEBU**	**IMLP**	**IMFV**	**IMCD**	**MEH**	**VALD**	**PUCA**	**CHIL**	**PIR**	**PM**	**CR**	**PARE**
	**LEBU**	—	0.000	0.000	0.000	0.169	0.000	0.000	0.887	0.000	0.000	0.428	0.070
	**IMLP**		—	0.992	0.049	0.000	0.944	0.373	0.000	1.000	0.901	0.000	0.000
	**IMFV**			—	0.640	0.000	1.000	0.982	0.000	0.980	0.238	0.000	0.000
	**IMCD**				—	0.000	0.854	0.999	0.000	0.028	0.000	0.000	0.000
	**MEH**					—	0.000	0.000	0.005	0.000	0.000	1.000	0.000
	**VALD**						—	0.999	0.000	0.899	0.111	0.000	0.000
	**PUCA**							—	0.000	0.275	0.003	0.000	0.000
	**CHIL**								—	0.000	0.000	0.001	0.758
	**PIR**									—	0.949	0.000	0.000
	**PM**										—	0.000	0.000
	**CR**											—	0.000
	**PARE**												—

**Table 5:** Hierarchical AMOVA of *Perumytilus purpuratus* when grouped using Bayesian inference (16S and 28S) and GENELAND clustering (28S) information; * *p* ≤ 0.01 is the probability that the observed values were equal to or smaller than those expected with random clustering; ns, non-significant.

Hierarchical Level	Source of Variation	df	Sum of Squares	Variance Components	Percentage of Variation	Fixation Indices
**16S clades**	Among groups	1	238.59	7.93	94.44	FCT = 0.94 *
	Among populations within groups	18	15.02	0.16	1.94	FST = 0.96 *
	Within populations	46	14.00	0.30	3.62	FSC = 0.35 *
	Total	65	267.61	8.40		
**28S clades**	Among groups	1	281.48	5.01	97.76	FCT = 0.98 *
	Among populations within groups	20	2.14	−0.002	−0.03	FST = 0.98 *
	Within populations	102	11.87	0.12	2.27	FSC = −0.02 ns
	Total	123	295.48	5.12		
**28S Clustering, K = 3**	Among groups	2	262.98	4.19	94.05	FCT = 0.94 *
	Among populations within groups	17	1.96	−0.03	−0.64	FST = −0.11 *
	Within populations	104	30.54	0.29	6.60	FSC = 0.93 ns
	Total	123	295.48	4.45		

**Table 6:** Genetic diversity indices based on mitochondrial (16s) and nuclear (28s) genes using morphologic (a) and genetic (b,c) groups as datasets. n, number of sequences; S, number of polymorphic sites; h, number of haplotypes; Hd, haplotype diversity; Pi, nucleotide diversity; K, average number of nucleotide differences.

	Mitochondrial rDNA 16s Gene						Nuclear rRNA 28s Gene					
**a. By Adjusted acrosome**	**n**	**S**	**h**	**Hd**	**Pi**	**K**	**n**	**S**	**h**	**Hd**	**Pi**	**K**
Short MPT	16	5	3	0.34	0.003	1.33	26	3	4	0.44	0.025	0.48
Intermediate MPT	19	21	9	0.68	0.009	3.61	39	10	5	0.32	0.124	1.98
Long MPT	19	7	7	0.54	0.002	0.83	38	3	3	0.10	0.009	0.16
**b. By BI clades**												
North clade	23	9	7	0.52	0.003	1.31	43	3	4	0.41	0.001	0.43
South clade	43	13	13	0.49	0.002	0.69	81	5	5	0.10	0.000	0.12
**c. By GENELAND clustering**												
Cluster 1: North	23	9	7	0.52	0.003	1.31	38	3	4	0.45	0.001	0.48
Cluster 2: Transition	-	-	-	-	-	-	8	8	2	0.54	0.007	4.29
Cluster 3: South	43	13	13	0.49	0.002	0.69	78	5	5	0.10	0.000	0.13
**All sequences**	66	33	20	0.73	0.018	7.31	124	15	9	0.55	0.006	3.89

**Table 7:** Neutrality tests performed for each clade from the mitochondrial 16s gene (M-type). Statistically significant results (*p* ≤ 0.01) are shown with an asterisk (*).

Dataset	Tajima’s D	Fu’s Fs	R2
North clade	−1.53	−2.2	0.07 *
South clade	−2.44 *	−14.07 *	0.04 *
All samples	−0.11	−1.14	0.11

## Data Availability

Data are contained within the article and Supplementary Material.

## Supplementary Materials

The following supporting information can be downloaded at: https://www.mdpi.com/article/10.3390/ani14050674/s1, Table S1. Results of linear regression of mean values by locality with latitude. Table S2. (A) ANOVA Post hoc Tukey test for the mean head length for all pairwise comparisons; Adjp is the adjusted *p*-value. 1 = Iquique, 2 =Antofagasta, 3 = Tumbes, 4 = Lota, 5 = Lebu, 6 = Isla Mocha Punta Los Piures, 7 = Isla Mocha Faro Viejo, 8 = Isla Mocha Caleta Derrumbe, 9 = Mehuín, 10 = Valdivia, 11 = Pucatrihue, 12 = Chiloé, 13 = Punta Pirámide, 14 = Puerto Madryn, 15 = Comodoro Rivadavia, 16 = Punta Arenas. (B) Matrix for pairwise comparisons of the adjusted *p*-value of the mean head length; sample codes are given in Table 1. Table S3. (A) ANCOVA Post hoc Tukey test for the mean acrosome length for all pairwise comparisons from the northern localities: 1 = Iquique, 2 = Antofagasta, 3 = Tumbes, 4 = Lota; Adjp is the adjusted *p*-value. (B) Least-square means of the acrosome lengths from northern localities. Sample codes are given in Table 1. Table S4. Model selection criteria (Akaike’s information criterion (AIC) and fitted parameters. AICc = Akaike’s adjusted for small sample sizes, Δ-AICc = difference between the AICc and the most parsimonious model’s AICc. *L =* Locality, *N* = Head, ** =* Interaction. Figure S1. Lollipop plot of the minimum and maximum values of the acrosome length, head length, and acrosome/head ratio by locality. The localities’ codes are shown in Table 1. Figure S2. Consensus tree from the Bayesian inference analysis. (A) Female clade (F-type) and (B) male clade (M-type) from the rRNA 16S haplotypes of *Perumytilus purpuratus*. Female (BRLGH) and male (BRLG) individuals of the outgroup species *Brachidontes rodriguezii* were included in this analysis. The localities’ codes are shown in Table 1. * Note: The MONT locality corresponds to VALP in Table 1.
