# Supplementary material for: Linking Acrosome Size and Genetic Divergence in an Inter-Oceanic Mussel from the Pacific and Atlantic Coasts: A Case of Incipient Speciation?
Source: Animals (Basel). 2024 Feb 21;14(5):674. doi: 10.3390/ani14050674 (PMC10930590; doi:10.3390/ani14050674)
Supplement: Supplementary file 1 [file animals-14-00674-s001.zip › animals-2843316-supplementary/Figure S2.pdf]

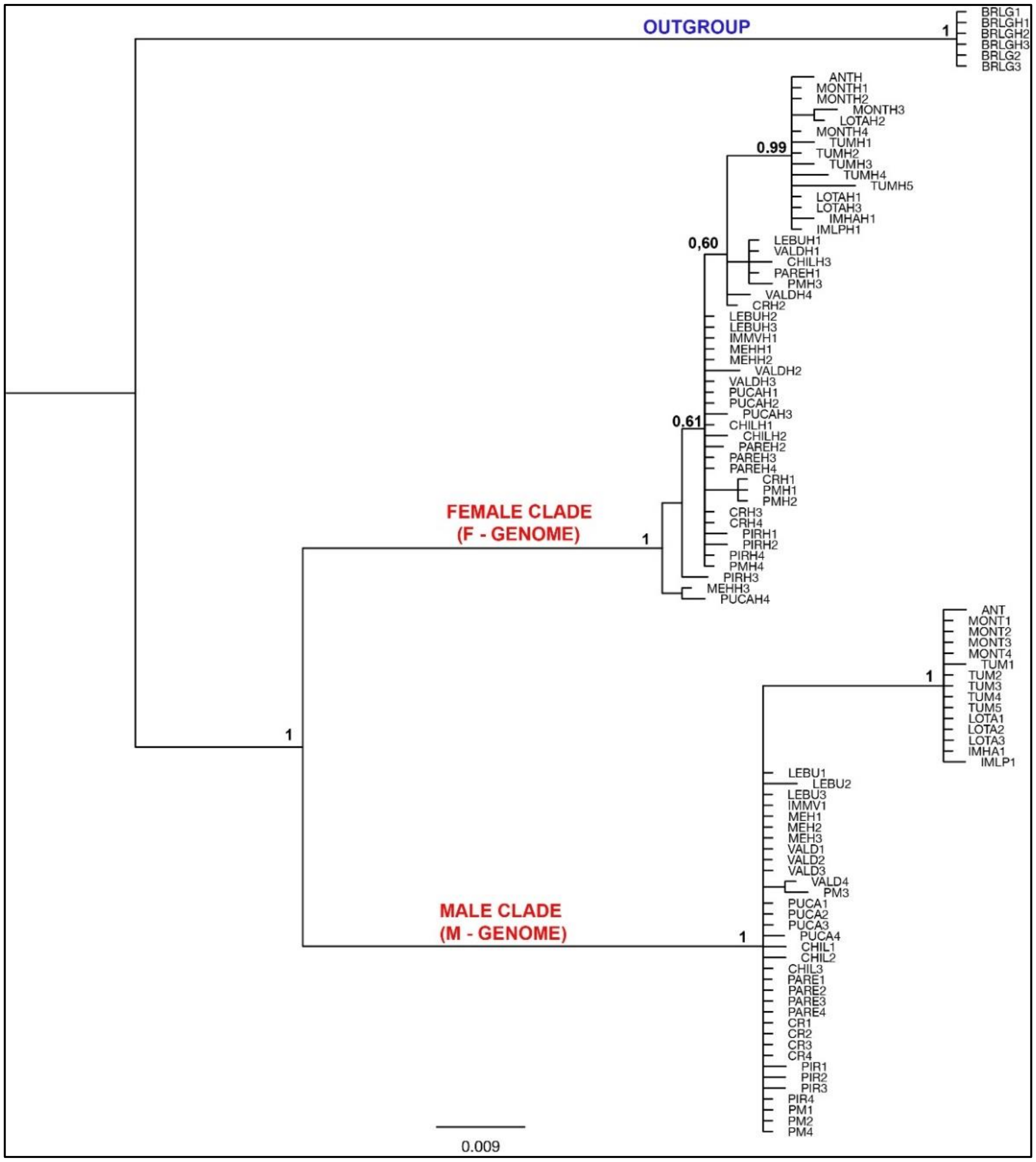

Figure S2. Consensus tree from the Bayesian inference analysis. **A)** Female clade (F-type) and **B)** male clade (M-type) from the rRNA 16S haplotypes of *Perumytilus purpuratus*. Female (BRLGH) and male (BRLG) individuals of the outgroup species *Brachidontes rodriguezii* were included in this analysis. The localities' codes are shown in Table 1. *Note: The MONT locality corresponds to VALP in Table 1.

Methods:

Bayesian Inference (BI) was performed using the MCMC method implemented in MrBayes 3.2.7 program. For this analysis 16S rRNA sequences from females and males were used. The outgroup species was *Brachidontes rodriguezii* sampled by us at Las Grutas, province de Neuquén, Argentina (40°48'S/65°05'W); the best-fit substitution model determinate by BIC in jModelTest v 2.1.8 was HKY + G. The BI analysis included two independent runs, four chains and a burn-in of 25% for a total of 5,000,000 generations sampled every 1000 generations. The resulting consensus tree was then visualised and edited in FigTree v 1.4.4.
