# Supplementary material for: Linking Acrosome Size and Genetic Divergence in an Inter-Oceanic Mussel from the Pacific and Atlantic Coasts: A Case of Incipient Speciation?
Source: Animals (Basel). 2024 Feb 21;14(5):674. doi: 10.3390/ani14050674 (PMC10930590; doi:10.3390/ani14050674)
Supplement: Supplementary file 1 [file animals-14-00674-s001.zip › animals-2843316-supplementary/Table S1.pdf]

Table S1. Results of linear regression of mean values by locality with latitude.

Norte

Acrosome length = 1.065 + 0.001 latitude, R^2 = 0.017, P = 0.869

Head length = 4.662 - 0.021 latitude, R^2 = 0.640, P = 0.199

Acrosome/Head ratio = 22.398 + latitude, R^2 = 0.124, P = 0.318

Sur

Traits

Acrosome length = 0.968 + 0.038 latitude, R^2 = 0.502, P = 0.010

Head length = 4.182 + 0.022 latitude, R^2 = 0.502, P = 0.257

Acrosome/Head ratio = 29.481 + 0.496 latitude, R^2 = 0.291, P = 0.070
