# Supplementary material for: Linking Acrosome Size and Genetic Divergence in an Inter-Oceanic Mussel from the Pacific and Atlantic Coasts: A Case of Incipient Speciation?
Source: Animals (Basel). 2024 Feb 21;14(5):674. doi: 10.3390/ani14050674 (PMC10930590; doi:10.3390/ani14050674)
Supplement: Supplementary file 1 [file animals-14-00674-s001.zip › animals-2843316-supplementary/Table S2.pdf]

Table S2.

A) Post hoc Tukey test for mean Acrosome length between all pair comparisons; Adjp is the adjusted p value. 1=Iquique, 2=Antofagasta, 3=Tumbes, 4=Lota, 5=Lebu, 6=Isla Mocha Punta los Piures, 7= Isla Mocha Faro Viejo, 8= Isla Mocha Caleta Derrumbe, 9= Mehuín, 10=Valdivia, 11=Pucatrihue, 12=Chiloé, 13=Punta Pirámide, 14=Puerto Madryn 15=Comodoro Rivadavia, 16=Punta Arenas.

NORD	Effect	NLOC	_NLOC	Estimate	StdErr	DF	tValue	Probt	Adjustment	Adjp
1	NLOC	1	2	11.4167	16.9172	464	0.67	0.5001	Tukey	1.0000
2	NLOC	1	3	19.4833	16.9172	464	1.15	0.2500	Tukey	0.9989
3	NLOC	1	4	-27.1667	16.9172	464	-1.61	0.1090	Tukey	0.9668
4	NLOC	1	5	-224.4000	16.9172	464	-13.26	0.0000	Tukey	0.0000
5	NLOC	1	6	-207.3333	16.9172	464	-12.26	0.0000	Tukey	0.0000
6	NLOC	1	7	-233.7000	16.9172	464	-13.81	0.0000	Tukey	0.0000
7	NLOC	1	8	-136.6667	16.9172	464	-8.08	0.0000	Tukey	0.0000
8	NLOC	1	9	-186.8167	16.9172	464	-11.04	0.0000	Tukey	0.0000
9	NLOC	1	10	-240.3333	16.9172	464	-14.21	0.0000	Tukey	0.0000
10	NLOC	1	11	-173.2000	16.9172	464	-10.24	0.0000	Tukey	0.0000
11	NLOC	1	12	-382.0167	16.9172	464	-22.58	0.0000	Tukey	0.0000
12	NLOC	1	13	-199.0667	16.9172	464	-11.77	0.0000	Tukey	0.0000
13	NLOC	1	14	-209.8167	16.9172	464	-12.40	0.0000	Tukey	0.0000
14	NLOC	1	15	-284.8500	16.9172	464	-16.84	0.0000	Tukey	0.0000
15	NLOC	1	16	-390.6000	16.9172	464	-23.09	0.0000	Tukey	0.0000
16	NLOC	2	3	8.0667	16.9172	464	0.48	0.6337	Tukey	1.0000
17	NLOC	2	4	-38.5833	16.9172	464	-2.28	0.0230	Tukey	0.6383
18	NLOC	2	5	-235.8167	16.9172	464	-13.94	0.0000	Tukey	0.0000
19	NLOC	2	6	-218.7500	16.9172	464	-12.93	0.0000	Tukey	0.0000
20	NLOC	2	7	-245.1167	16.9172	464	-14.49	0.0000	Tukey	0.0000
21	NLOC	2	8	-148.0833	16.9172	464	-8.75	0.0000	Tukey	0.0000
22	NLOC	2	9	-198.2333	16.9172	464	-11.72	0.0000	Tukey	0.0000
23	NLOC	2	10	-251.7500	16.9172	464	-14.88	0.0000	Tukey	0.0000
24	NLOC	2	11	-184.6167	16.9172	464	-10.91	0.0000	Tukey	0.0000
25	NLOC	2	12	-393.4333	16.9172	464	-23.26	0.0000	Tukey	0.0000
26	NLOC	2	13	-210.4833	16.9172	464	-12.44	0.0000	Tukey	0.0000
27	NLOC	2	14	-221.2333	16.9172	464	-13.08	0.0000	Tukey	0.0000
28	NLOC	2	15	-296.2667	16.9172	464	-17.51	0.0000	Tukey	0.0000
29	NLOC	2	16	-402.0167	16.9172	464	-23.76	0.0000	Tukey	0.0000
30	NLOC	3	4	-46.6500	16.9172	464	-2.76	0.0061	Tukey	0.2978
31	NLOC	3	5	-243.8833	16.9172	464	-14.42	0.0000	Tukey	0.0000
32	NLOC	3	6	-226.8167	16.9172	464	-13.41	0.0000	Tukey	0.0000
33	NLOC	3	7	-253.1833	16.9172	464	-14.97	0.0000	Tukey	0.0000
34	NLOC	3	8	-156.1500	16.9172	464	-9.23	0.0000	Tukey	0.0000
35	NLOC	3	9	-206.3000	16.9172	464	-12.19	0.0000	Tukey	0.0000

36	NLOC	3	10	-259.8167	16.9172	464	-15.36	0.0000	Tukey	0.0000
37	NLOC	3	11	-192.6833	16.9172	464	-11.39	0.0000	Tukey	0.0000
38	NLOC	3	12	-401.5000	16.9172	464	-23.73	0.0000	Tukey	0.0000
39	NLOC	3	13	-218.5500	16.9172	464	-12.92	0.0000	Tukey	0.0000
40	NLOC	3	14	-229.3000	16.9172	464	-13.55	0.0000	Tukey	0.0000
41	NLOC	3	15	-304.3333	16.9172	464	-17.99	0.0000	Tukey	0.0000
42	NLOC	3	16	-410.0833	16.9172	464	-24.24	0.0000	Tukey	0.0000
43	NLOC	4	5	-197.2333	16.9172	464	-11.66	0.0000	Tukey	0.0000
44	NLOC	4	6	-180.1667	16.9172	464	-10.65	0.0000	Tukey	0.0000
45	NLOC	4	7	-206.5333	16.9172	464	-12.21	0.0000	Tukey	0.0000
46	NLOC	4	8	-109.5000	16.9172	464	-6.47	0.0000	Tukey	0.0000
47	NLOC	4	9	-159.6500	16.9172	464	-9.44	0.0000	Tukey	0.0000
48	NLOC	4	10	-213.1667	16.9172	464	-12.60	0.0000	Tukey	0.0000
49	NLOC	4	11	-146.0333	16.9172	464	-8.63	0.0000	Tukey	0.0000
50	NLOC	4	12	-354.8500	16.9172	464	-20.98	0.0000	Tukey	0.0000
51	NLOC	4	13	-171.9000	16.9172	464	-10.16	0.0000	Tukey	0.0000
52	NLOC	4	14	-182.6500	16.9172	464	-10.80	0.0000	Tukey	0.0000
53	NLOC	4	15	-257.6833	16.9172	464	-15.23	0.0000	Tukey	0.0000
54	NLOC	4	16	-363.4333	16.9172	464	-21.48	0.0000	Tukey	0.0000
55	NLOC	5	6	17.0667	16.9172	464	1.01	0.3136	Tukey	0.9998
56	NLOC	5	7	-9.3000	16.9172	464	-0.55	0.5828	Tukey	1.0000
57	NLOC	5	8	87.7333	16.9172	464	5.19	0.0000	Tukey	0.0000
58	NLOC	5	9	37.5833	16.9172	464	2.22	0.0268	Tukey	0.6814
59	NLOC	5	10	-15.9333	16.9172	464	-0.94	0.3468	Tukey	0.9999
60	NLOC	5	11	51.2000	16.9172	464	3.03	0.0026	Tukey	0.1620
61	NLOC	5	12	-157.6167	16.9172	464	-9.32	0.0000	Tukey	0.0000
62	NLOC	5	13	25.3333	16.9172	464	1.50	0.1349	Tukey	0.9825
63	NLOC	5	14	14.5833	16.9172	464	0.86	0.3891	Tukey	1.0000
64	NLOC	5	15	-60.4500	16.9172	464	-3.57	0.0004	Tukey	0.0332
65	NLOC	5	16	-166.2000	16.9172	464	-9.82	0.0000	Tukey	0.0000
66	NLOC	6	7	-26.3667	16.9172	464	-1.56	0.1198	Tukey	0.9746
67	NLOC	6	8	70.6667	16.9172	464	4.18	0.0000	Tukey	0.0036
68	NLOC	6	9	20.5167	16.9172	464	1.21	0.2258	Tukey	0.9980
69	NLOC	6	10	-33.0000	16.9172	464	-1.95	0.0517	Tukey	0.8505
70	NLOC	6	11	34.1333	16.9172	464	2.02	0.0442	Tukey	0.8144
71	NLOC	6	12	-174.6833	16.9172	464	-10.33	0.0000	Tukey	0.0000
72	NLOC	6	13	8.2667	16.9172	464	0.49	0.6253	Tukey	1.0000
73	NLOC	6	14	-2.4833	16.9172	464	-0.15	0.8834	Tukey	1.0000
74	NLOC	6	15	-77.5167	16.9172	464	-4.58	0.0000	Tukey	0.0007
75	NLOC	6	16	-183.2667	16.9172	464	-10.83	0.0000	Tukey	0.0000
76	NLOC	7	8	97.0333	16.9172	464	5.74	0.0000	Tukey	0.0000
77	NLOC	7	9	46.8833	16.9172	464	2.77	0.0058	Tukey	0.2895
78	NLOC	7	10	-6.6333	16.9172	464	-0.39	0.6952	Tukey	1.0000

79	NLOC	7	11	60.5000	16.9172	464	3.58	0.0004	Tukey	0.0328
80	NLOC	7	12	-148.3167	16.9172	464	-8.77	0.0000	Tukey	0.0000
81	NLOC	7	13	34.6333	16.9172	464	2.05	0.0412	Tukey	0.7971
82	NLOC	7	14	23.8833	16.9172	464	1.41	0.1587	Tukey	0.9901
83	NLOC	7	15	-51.1500	16.9172	464	-3.02	0.0026	Tukey	0.1632
84	NLOC	7	16	-156.9000	16.9172	464	-9.27	0.0000	Tukey	0.0000
85	NLOC	8	9	-50.1500	16.9172	464	-2.96	0.0032	Tukey	0.1885
86	NLOC	8	10	-103.6667	16.9172	464	-6.13	0.0000	Tukey	0.0000
87	NLOC	8	11	-36.5333	16.9172	464	-2.16	0.0313	Tukey	0.7249
88	NLOC	8	12	-245.3500	16.9172	464	-14.50	0.0000	Tukey	0.0000
89	NLOC	8	13	-62.4000	16.9172	464	-3.69	0.0003	Tukey	0.0225
90	NLOC	8	14	-73.1500	16.9172	464	-4.32	0.0000	Tukey	0.0020
91	NLOC	8	15	-148.1833	16.9172	464	-8.76	0.0000	Tukey	0.0000
92	NLOC	8	16	-253.9333	16.9172	464	-15.01	0.0000	Tukey	0.0000
93	NLOC	9	10	-53.5167	16.9172	464	-3.16	0.0017	Tukey	0.1136
94	NLOC	9	11	13.6167	16.9172	464	0.80	0.4213	Tukey	1.0000
95	NLOC	9	12	-195.2000	16.9172	464	-11.54	0.0000	Tukey	0.0000
96	NLOC	9	13	-12.2500	16.9172	464	-0.72	0.4694	Tukey	1.0000
97	NLOC	9	14	-23.0000	16.9172	464	-1.36	0.1746	Tukey	0.9933
98	NLOC	9	15	-98.0333	16.9172	464	-5.79	0.0000	Tukey	0.0000
99	NLOC	9	16	-203.7833	16.9172	464	-12.05	0.0000	Tukey	0.0000
100	NLOC	10	11	67.1333	16.9172	464	3.97	0.0001	Tukey	0.0082
101	NLOC	10	12	-141.6833	16.9172	464	-8.38	0.0000	Tukey	0.0000
102	NLOC	10	13	41.2667	16.9172	464	2.44	0.0151	Tukey	0.5188
103	NLOC	10	14	30.5167	16.9172	464	1.80	0.0719	Tukey	0.9141
104	NLOC	10	15	-44.5167	16.9172	464	-2.63	0.0088	Tukey	0.3793
105	NLOC	10	16	-150.2667	16.9172	464	-8.88	0.0000	Tukey	0.0000
106	NLOC	11	12	-208.8167	16.9172	464	-12.34	0.0000	Tukey	0.0000
107	NLOC	11	13	-25.8667	16.9172	464	-1.53	0.1269	Tukey	0.9787
108	NLOC	11	14	-36.6167	16.9172	464	-2.16	0.0309	Tukey	0.7216
109	NLOC	11	15	-111.6500	16.9172	464	-6.60	0.0000	Tukey	0.0000
110	NLOC	11	16	-217.4000	16.9172	464	-12.85	0.0000	Tukey	0.0000
111	NLOC	12	13	182.9500	16.9172	464	10.81	0.0000	Tukey	0.0000
112	NLOC	12	14	172.2000	16.9172	464	10.18	0.0000	Tukey	0.0000
113	NLOC	12	15	97.1667	16.9172	464	5.74	0.0000	Tukey	0.0000
114	NLOC	12	16	-8.5833	16.9172	464	-0.51	0.6121	Tukey	1.0000
115	NLOC	13	14	-10.7500	16.9172	464	-0.64	0.5255	Tukey	1.0000
116	NLOC	13	15	-85.7833	16.9172	464	-5.07	0.0000	Tukey	0.0001
117	NLOC	13	16	-191.5333	16.9172	464	-11.32	0.0000	Tukey	0.0000
118	NLOC	14	15	-75.0333	16.9172	464	-4.44	0.0000	Tukey	0.0012
119	NLOC	14	16	-180.7833	16.9172	464	-10.69	0.0000	Tukey	0.0000
120	NLOC	15	16	-105.7500	16.9172	464	-6.25	0.0000	Tukey	0.0000

B) Matrix for pairwise comparisons of the Adjusted P value of mean Acrosome length. Sample codes in Table 1

Locality	IQQ	ANT	TUMB	LOTA	LEBU	IMLP	IMFV	IMCD	MEH	VALD	PUCA	CHIL	PIR	PM	CR	PARE
IQQ	1	1.0000	0.9989	0.9668	0.0000	0.0000	0.0000	0.0000	0.0000	0.0000	0.0000	0.0000	0.0000	0.0000	0.0000	0.0000
ANT	1.0000	1	1.0000	0.6383	0.0000	0.0000	0.0000	0.0000	0.0000	0.0000	0.0000	0.0000	0.0000	0.0000	0.0000	0.0000
TUMB	0.9989	1.0000	1	0.2978	0.0000	0.0000	0.0000	0.0000	0.0000	0.0000	0.0000	0.0000	0.0000	0.0000	0.0000	0.0000
LOTA	0.9668	0.6383	0.2978	1	0.0000	0.0000	0.0000	0.0000	0.0000	0.0000	0.0000	0.0000	0.0000	0.0000	0.0000	0.0000
LEBU	0.0000	0.0000	0.0000	0.0000	1	0.9998	1.0000	0.0000	0.6814	0.9999	0.1620	0.0000	0.9825	1.0000	0.0332	0.0000
IMLP	0.0000	0.0000	0.0000	0.0000	0.9998	1	0.9746	0.0036	0.9980	0.8505	0.8144	0.0000	1.0000	1.0000	0.0007	0.0000
IMFV	0.0000	0.0000	0.0000	0.0000	1.0000	0.9746	1	0.0000	0.2895	1.0000	0.0328	0.0000	0.7971	0.9901	0.1632	0.0000
IMCD	0.0000	0.0000	0.0000	0.0000	0.0000	0.0036	0.0000	1	0.1885	0.0000	0.7249	0.0000	0.0225	0.0020	0.0000	0.0000
MEH	0.0000	0.0000	0.0000	0.0000	0.6814	0.9980	0.2895	0.1885	1	0.1136	1.0000	0.0000	1.0000	0.9933	0.0000	0.0000
VALD	0.0000	0.0000	0.0000	0.0000	0.9999	0.8505	1.0000	0.0000	0.1136	1	0.0082	0.0000	0.5188	0.9141	0.3793	0.0000
PUCA	0.0000	0.0000	0.0000	0.0000	0.1620	0.8144	0.0328	0.7249	1.0000	0.0082	1	0.0000	0.9787	0.7216	0.0000	0.0000
CHIL	0.0000	0.0000	0.0000	0.0000	0.0000	0.0000	0.0000	0.0000	0.0000	0.0000	0.0000	1	0.0000	0.0000	0.0000	1.0000
PIR	0.0000	0.0000	0.0000	0.0000	0.9825	1.0000	0.7971	0.0225	1.0000	0.5188	0.9787	0.0000	1	1.0000	0.0001	0.0000
PM	0.0000	0.0000	0.0000	0.0000	1.0000	1.0000	0.9901	0.0020	0.9933	0.9141	0.7216	0.0000	1.0000	1	0.0012	0.0000
CR	0.0000	0.0000	0.0000	0.0000	0.0332	0.0007	0.1632	0.0000	0.0000	0.3793	0.0000	0.0000	0.0001	0.0012	1	0.0000
PARE	0.0000	0.0000	0.0000	0.0000	0.0000	0.0000	0.0000	0.0000	0.0000	0.0000	0.0000	1.0000	0.0000	0.0000	0.0000	1
