# Supplementary material for: Linking Acrosome Size and Genetic Divergence in an Inter-Oceanic Mussel from the Pacific and Atlantic Coasts: A Case of Incipient Speciation?
Source: Animals (Basel). 2024 Feb 21;14(5):674. doi: 10.3390/ani14050674 (PMC10930590; doi:10.3390/ani14050674)
Supplement: Supplementary file 1 [file animals-14-00674-s001.zip › animals-2843316-supplementary/Table S3.pdf]

**Table S**3.

A) Post hoc Tukey test for mean Head length between all pair comparisons; Adjp is the adjusted p value. 1=Iquique, 2=Antofagasta, 3=Tumbes, 4=Lota, 5=Lebu, 6=Isla Mocha Punta los Piures, 7= Isla Mocha Faro Viejo, 8= Isla Mocha Caleta Derrumbe, 9= Mehuín, 10=Valdivia, 11=Pucatrihue, 12=Chiloé, 13=Punta Pirámide, 14=Puerto Madryn 15=Comodoro Rivadavia, 16=Punta Arenas.

| NORD | Effect | NLOC | _NLOC | Estimate | StdErr | DF | tValue | Probt | Adjustment | Adjp |
|---|---|---|---|---|---|---|---|---|---|---|
| 1 | NLOC | 1 | 2 | 62.8167 | 15.8861 | 464 | 3.95 | 0.0001 | Tukey | 0.0086 |
| 2 | NLOC | 1 | 3 | 43.3167 | 15.8861 | 464 | 2.73 | 0.0066 | Tukey | 0.3168 |
| 3 | NLOC | 1 | 4 | 43.9667 | 15.8861 | 464 | 2.77 | 0.0059 | Tukey | 0.2917 |
| 4 | NLOC | 1 | 5 | -50.1833 | 15.8861 | 464 | -3.16 | 0.0017 | Tukey | 0.1150 |
| 5 | NLOC | 1 | 6 | -211.8167 | 15.8861 | 464 | -13.33 | 0.0000 | Tukey | 0.0000 |
| 6 | NLOC | 1 | 7 | -270.5833 | 15.8861 | 464 | -17.03 | 0.0000 | Tukey | 0.0000 |
| 7 | NLOC | 1 | 8 | -175.8667 | 15.8861 | 464 | -11.07 | 0.0000 | Tukey | 0.0000 |
| 8 | NLOC | 1 | 9 | -63.4500 | 15.8861 | 464 | -3.99 | 0.0001 | Tukey | 0.0074 |
| 9 | NLOC | 1 | 10 | -298.6000 | 15.8861 | 464 | -18.80 | 0.0000 | Tukey | 0.0000 |
| 10 | NLOC | 1 | 11 | -209.3667 | 15.8861 | 464 | -13.18 | 0.0000 | Tukey | 0.0000 |
| 11 | NLOC | 1 | 12 | -300.3667 | 15.8861 | 464 | -18.91 | 0.0000 | Tukey | 0.0000 |
| 12 | NLOC | 1 | 13 | -195.4167 | 15.8861 | 464 | -12.30 | 0.0000 | Tukey | 0.0000 |
| 13 | NLOC | 1 | 14 | -178.4000 | 15.8861 | 464 | -11.23 | 0.0000 | Tukey | 0.0000 |
| 14 | NLOC | 1 | 15 | -162.6667 | 15.8861 | 464 | -10.24 | 0.0000 | Tukey | 0.0000 |
| 15 | NLOC | 1 | 16 | -289.3833 | 15.8861 | 464 | -18.22 | 0.0000 | Tukey | 0.0000 |
| 16 | NLOC | 2 | 3 | -19.5000 | 15.8861 | 464 | -1.23 | 0.2203 | Tukey | 0.9977 |
| 17 | NLOC | 2 | 4 | -18.8500 | 15.8861 | 464 | -1.19 | 0.2360 | Tukey | 0.9985 |
| 18 | NLOC | 2 | 5 | -113.0000 | 15.8861 | 464 | -7.11 | 0.0000 | Tukey | 0.0000 |
| 19 | NLOC | 2 | 6 | -274.6333 | 15.8861 | 464 | -17.29 | 0.0000 | Tukey | 0.0000 |
| 20 | NLOC | 2 | 7 | -333.4000 | 15.8861 | 464 | -20.99 | 0.0000 | Tukey | 0.0000 |
| 21 | NLOC | 2 | 8 | -238.6833 | 15.8861 | 464 | -15.02 | 0.0000 | Tukey | 0.0000 |
| 22 | NLOC | 2 | 9 | -126.2667 | 15.8861 | 464 | -7.95 | 0.0000 | Tukey | 0.0000 |
| 23 | NLOC | 2 | 10 | -361.4167 | 15.8861 | 464 | -22.75 | 0.0000 | Tukey | 0.0000 |
| 24 | NLOC | 2 | 11 | -272.1833 | 15.8861 | 464 | -17.13 | 0.0000 | Tukey | 0.0000 |
| 25 | NLOC | 2 | 12 | -363.1833 | 15.8861 | 464 | -22.86 | 0.0000 | Tukey | 0.0000 |
| 26 | NLOC | 2 | 13 | -258.2333 | 15.8861 | 464 | -16.26 | 0.0000 | Tukey | 0.0000 |
| 27 | NLOC | 2 | 14 | -241.2167 | 15.8861 | 464 | -15.18 | 0.0000 | Tukey | 0.0000 |
| 28 | NLOC | 2 | 15 | -225.4833 | 15.8861 | 464 | -14.19 | 0.0000 | Tukey | 0.0000 |
| 29 | NLOC | 2 | 16 | -352.2000 | 15.8861 | 464 | -22.17 | 0.0000 | Tukey | 0.0000 |
| 30 | NLOC | 3 | 4 | 0.6500 | 15.8861 | 464 | 0.04 | 0.9674 | Tukey | 1.0000 |
| 31 | NLOC | 3 | 5 | -93.5000 | 15.8861 | 464 | -5.89 | 0.0000 | Tukey | 0.0000 |
| 32 | NLOC | 3 | 6 | -255.1333 | 15.8861 | 464 | -16.06 | 0.0000 | Tukey | 0.0000 |
| 33 | NLOC | 3 | 7 | -313.9000 | 15.8861 | 464 | -19.76 | 0.0000 | Tukey | 0.0000 |
| 34 | NLOC | 3 | 8 | -219.1833 | 15.8861 | 464 | -13.80 | 0.0000 | Tukey | 0.0000 |
| 35 | NLOC | 3 | 9 | -106.7667 | 15.8861 | 464 | -6.72 | 0.0000 | Tukey | 0.0000 |
| 36 | NLOC | 3 | 10 | -341.9167 | 15.8861 | 464 | -21.52 | 0.0000 | Tukey | 0.0000 |

| | | | | | | | | | | |
|---|---|---|---|---|---|---|---|---|---|---|
| 37 | NLOC | 3 | 11 | -252.6833 | 15.8861 | 464 | -15.91 | 0.0000 | Tukey | 0.0000 |
| 38 | NLOC | 3 | 12 | -343.6833 | 15.8861 | 464 | -21.63 | 0.0000 | Tukey | 0.0000 |
| 39 | NLOC | 3 | 13 | -238.7333 | 15.8861 | 464 | -15.03 | 0.0000 | Tukey | 0.0000 |
| 40 | NLOC | 3 | 14 | -221.7167 | 15.8861 | 464 | -13.96 | 0.0000 | Tukey | 0.0000 |
| 41 | NLOC | 3 | 15 | -205.9833 | 15.8861 | 464 | -12.97 | 0.0000 | Tukey | 0.0000 |
| 42 | NLOC | 3 | 16 | -332.7000 | 15.8861 | 464 | -20.94 | 0.0000 | Tukey | 0.0000 |
| 43 | NLOC | 4 | 5 | -94.1500 | 15.8861 | 464 | -5.93 | 0.0000 | Tukey | 0.0000 |
| 44 | NLOC | 4 | 6 | -255.7833 | 15.8861 | 464 | -16.10 | 0.0000 | Tukey | 0.0000 |
| 45 | NLOC | 4 | 7 | -314.5500 | 15.8861 | 464 | -19.80 | 0.0000 | Tukey | 0.0000 |
| 46 | NLOC | 4 | 8 | -219.8333 | 15.8861 | 464 | -13.84 | 0.0000 | Tukey | 0.0000 |
| 47 | NLOC | 4 | 9 | -107.4167 | 15.8861 | 464 | -6.76 | 0.0000 | Tukey | 0.0000 |
| 48 | NLOC | 4 | 10 | -342.5667 | 15.8861 | 464 | -21.56 | 0.0000 | Tukey | 0.0000 |
| 49 | NLOC | 4 | 11 | -253.3333 | 15.8861 | 464 | -15.95 | 0.0000 | Tukey | 0.0000 |
| 50 | NLOC | 4 | 12 | -344.3333 | 15.8861 | 464 | -21.68 | 0.0000 | Tukey | 0.0000 |
| 51 | NLOC | 4 | 13 | -239.3833 | 15.8861 | 464 | -15.07 | 0.0000 | Tukey | 0.0000 |
| 52 | NLOC | 4 | 14 | -222.3667 | 15.8861 | 464 | -14.00 | 0.0000 | Tukey | 0.0000 |
| 53 | NLOC | 4 | 15 | -206.6333 | 15.8861 | 464 | -13.01 | 0.0000 | Tukey | 0.0000 |
| 54 | NLOC | 4 | 16 | -333.3500 | 15.8861 | 464 | -20.98 | 0.0000 | Tukey | 0.0000 |
| 55 | NLOC | 5 | 6 | -161.6333 | 15.8861 | 464 | -10.17 | 0.0000 | Tukey | 0.0000 |
| 56 | NLOC | 5 | 7 | -220.4000 | 15.8861 | 464 | -13.87 | 0.0000 | Tukey | 0.0000 |
| 57 | NLOC | 5 | 8 | -125.6833 | 15.8861 | 464 | -7.91 | 0.0000 | Tukey | 0.0000 |
| 58 | NLOC | 5 | 9 | -13.2667 | 15.8861 | 464 | -0.84 | 0.4041 | Tukey | 1.0000 |
| 59 | NLOC | 5 | 10 | -248.4167 | 15.8861 | 464 | -15.64 | 0.0000 | Tukey | 0.0000 |
| 60 | NLOC | 5 | 11 | -159.1833 | 15.8861 | 464 | -10.02 | 0.0000 | Tukey | 0.0000 |
| 61 | NLOC | 5 | 12 | -250.1833 | 15.8861 | 464 | -15.75 | 0.0000 | Tukey | 0.0000 |
| 62 | NLOC | 5 | 13 | -145.2333 | 15.8861 | 464 | -9.14 | 0.0000 | Tukey | 0.0000 |
| 63 | NLOC | 5 | 14 | -128.2167 | 15.8861 | 464 | -8.07 | 0.0000 | Tukey | 0.0000 |
| 64 | NLOC | 5 | 15 | -112.4833 | 15.8861 | 464 | -7.08 | 0.0000 | Tukey | 0.0000 |
| 65 | NLOC | 5 | 16 | -239.2000 | 15.8861 | 464 | -15.06 | 0.0000 | Tukey | 0.0000 |
| 66 | NLOC | 6 | 7 | -58.7667 | 15.8861 | 464 | -3.70 | 0.0002 | Tukey | 0.0217 |
| 67 | NLOC | 6 | 8 | 35.9500 | 15.8861 | 464 | 2.26 | 0.0241 | Tukey | 0.6514 |
| 68 | NLOC | 6 | 9 | 148.3667 | 15.8861 | 464 | 9.34 | 0.0000 | Tukey | 0.0000 |
| 69 | NLOC | 6 | 10 | -86.7833 | 15.8861 | 464 | -5.46 | 0.0000 | Tukey | 0.0000 |
| 70 | NLOC | 6 | 11 | 2.4500 | 15.8861 | 464 | 0.15 | 0.8775 | Tukey | 1.0000 |
| 71 | NLOC | 6 | 12 | -88.5500 | 15.8861 | 464 | -5.57 | 0.0000 | Tukey | 0.0000 |
| 72 | NLOC | 6 | 13 | 16.4000 | 15.8861 | 464 | 1.03 | 0.3024 | Tukey | 0.9997 |
| 73 | NLOC | 6 | 14 | 33.4167 | 15.8861 | 464 | 2.10 | 0.0360 | Tukey | 0.7621 |
| 74 | NLOC | 6 | 15 | 49.1500 | 15.8861 | 464 | 3.09 | 0.0021 | Tukey | 0.1365 |
| 75 | NLOC | 6 | 16 | -77.5667 | 15.8861 | 464 | -4.88 | 0.0000 | Tukey | 0.0002 |
| 76 | NLOC | 7 | 8 | 94.7167 | 15.8861 | 464 | 5.96 | 0.0000 | Tukey | 0.0000 |
| 77 | NLOC | 7 | 9 | 207.1333 | 15.8861 | 464 | 13.04 | 0.0000 | Tukey | 0.0000 |
| 78 | NLOC | 7 | 10 | -28.0167 | 15.8861 | 464 | -1.76 | 0.0785 | Tukey | 0.9278 |
| 79 | NLOC | 7 | 11 | 61.2167 | 15.8861 | 464 | 3.85 | 0.0001 | Tukey | 0.0125 |

| | | | | | | | | | | |
|---|---|---|---|---|---|---|---|---|---|---|
| 80 | NLOC | 7 | 12 | -29.7833 | 15.8861 | 464 | -1.87 | 0.0614 | Tukey | 0.8861 |
| 81 | NLOC | 7 | 13 | 75.1667 | 15.8861 | 464 | 4.73 | 0.0000 | Tukey | 0.0003 |
| 82 | NLOC | 7 | 14 | 92.1833 | 15.8861 | 464 | 5.80 | 0.0000 | Tukey | 0.0000 |
| 83 | NLOC | 7 | 15 | 107.9167 | 15.8861 | 464 | 6.79 | 0.0000 | Tukey | 0.0000 |
| 84 | NLOC | 7 | 16 | -18.8000 | 15.8861 | 464 | -1.18 | 0.2372 | Tukey | 0.9985 |
| 85 | NLOC | 8 | 9 | 112.4167 | 15.8861 | 464 | 7.08 | 0.0000 | Tukey | 0.0000 |
| 86 | NLOC | 8 | 10 | -122.7333 | 15.8861 | 464 | -7.73 | 0.0000 | Tukey | 0.0000 |
| 87 | NLOC | 8 | 11 | -33.5000 | 15.8861 | 464 | -2.11 | 0.0355 | Tukey | 0.7587 |
| 88 | NLOC | 8 | 12 | -124.5000 | 15.8861 | 464 | -7.84 | 0.0000 | Tukey | 0.0000 |
| 89 | NLOC | 8 | 13 | -19.5500 | 15.8861 | 464 | -1.23 | 0.2191 | Tukey | 0.9977 |
| 90 | NLOC | 8 | 14 | -2.5333 | 15.8861 | 464 | -0.16 | 0.8734 | Tukey | 1.0000 |
| 91 | NLOC | 8 | 15 | 13.2000 | 15.8861 | 464 | 0.83 | 0.4064 | Tukey | 1.0000 |
| 92 | NLOC | 8 | 16 | -113.5167 | 15.8861 | 464 | -7.15 | 0.0000 | Tukey | 0.0000 |
| 93 | NLOC | 9 | 10 | -235.1500 | 15.8861 | 464 | -14.80 | 0.0000 | Tukey | 0.0000 |
| 94 | NLOC | 9 | 11 | -145.9167 | 15.8861 | 464 | -9.19 | 0.0000 | Tukey | 0.0000 |
| 95 | NLOC | 9 | 12 | -236.9167 | 15.8861 | 464 | -14.91 | 0.0000 | Tukey | 0.0000 |
| 96 | NLOC | 9 | 13 | -131.9667 | 15.8861 | 464 | -8.31 | 0.0000 | Tukey | 0.0000 |
| 97 | NLOC | 9 | 14 | -114.9500 | 15.8861 | 464 | -7.24 | 0.0000 | Tukey | 0.0000 |
| 98 | NLOC | 9 | 15 | -99.2167 | 15.8861 | 464 | -6.25 | 0.0000 | Tukey | 0.0000 |
| 99 | NLOC | 9 | 16 | -225.9333 | 15.8861 | 464 | -14.22 | 0.0000 | Tukey | 0.0000 |
| 100 | NLOC | 10 | 11 | 89.2333 | 15.8861 | 464 | 5.62 | 0.0000 | Tukey | 0.0000 |
| 101 | NLOC | 10 | 12 | -1.7667 | 15.8861 | 464 | -0.11 | 0.9115 | Tukey | 1.0000 |
| 102 | NLOC | 10 | 13 | 103.1833 | 15.8861 | 464 | 6.50 | 0.0000 | Tukey | 0.0000 |
| 103 | NLOC | 10 | 14 | 120.2000 | 15.8861 | 464 | 7.57 | 0.0000 | Tukey | 0.0000 |
| 104 | NLOC | 10 | 15 | 135.9333 | 15.8861 | 464 | 8.56 | 0.0000 | Tukey | 0.0000 |
| 105 | NLOC | 10 | 16 | 9.2167 | 15.8861 | 464 | 0.58 | 0.5621 | Tukey | 1.0000 |
| 106 | NLOC | 11 | 12 | -91.0000 | 15.8861 | 464 | -5.73 | 0.0000 | Tukey | 0.0000 |
| 107 | NLOC | 11 | 13 | 13.9500 | 15.8861 | 464 | 0.88 | 0.3803 | Tukey | 1.0000 |
| 108 | NLOC | 11 | 14 | 30.9667 | 15.8861 | 464 | 1.95 | 0.0519 | Tukey | 0.8512 |
| 109 | NLOC | 11 | 15 | 46.7000 | 15.8861 | 464 | 2.94 | 0.0034 | Tukey | 0.1999 |
| 110 | NLOC | 11 | 16 | -80.0167 | 15.8861 | 464 | -5.04 | 0.0000 | Tukey | 0.0001 |
| 111 | NLOC | 12 | 13 | 104.9500 | 15.8861 | 464 | 6.61 | 0.0000 | Tukey | 0.0000 |
| 112 | NLOC | 12 | 14 | 121.9667 | 15.8861 | 464 | 7.68 | 0.0000 | Tukey | 0.0000 |
| 113 | NLOC | 12 | 15 | 137.7000 | 15.8861 | 464 | 8.67 | 0.0000 | Tukey | 0.0000 |
| 114 | NLOC | 12 | 16 | 10.9833 | 15.8861 | 464 | 0.69 | 0.4897 | Tukey | 1.0000 |
| 115 | NLOC | 13 | 14 | 17.0167 | 15.8861 | 464 | 1.07 | 0.2847 | Tukey | 0.9995 |
| 116 | NLOC | 13 | 15 | 32.7500 | 15.8861 | 464 | 2.06 | 0.0398 | Tukey | 0.7884 |
| 117 | NLOC | 13 | 16 | -93.9667 | 15.8861 | 464 | -5.92 | 0.0000 | Tukey | 0.0000 |
| 118 | NLOC | 14 | 15 | 15.7333 | 15.8861 | 464 | 0.99 | 0.3225 | Tukey | 0.9998 |
| 119 | NLOC | 14 | 16 | -110.9833 | 15.8861 | 464 | -6.99 | 0.0000 | Tukey | 0.0000 |
| 120 | NLOC | 15 | 16 | -126.7167 | 15.8861 | 464 | -7.98 | 0.0000 | Tukey | 0.0000 |

**B)** Matrix for pairwise comparisons of the Adjusted P value of mean Head length. Sample codes in Table 1

| Locality | IQQ | ANT | TUMB | LOTA | LEBU | IMLP | IMFV | IMCD | MEH | VALD | PUCA | CHIL | PIR | PM | CR | PARE |
|---|---|---|---|---|---|---|---|---|---|---|---|---|---|---|---|---|
| IQQ | 1 | 0.0086 | 0.3168 | 0.2917 | 0.1150 | 0.0000 | 0.0000 | 0.0000 | 0.0074 | 0.0000 | 0.0000 | 0.0000 | 0.0000 | 0.0000 | 0.0000 | 0.0000 |
| ANT | 0.0086 | 1 | 0.9977 | 0.9985 | 0.0000 | 0.0000 | 0.0000 | 0.0000 | 0.0000 | 0.0000 | 0.0000 | 0.0000 | 0.0000 | 0.0000 | 0.0000 | 0.0000 |
| TUMB | 0.3168 | 0.9977 | 1 | 1.0000 | 0.0000 | 0.0000 | 0.0000 | 0.0000 | 0.0000 | 0.0000 | 0.0000 | 0.0000 | 0.0000 | 0.0000 | 0.0000 | 0.0000 |
| LOTA | 0.2917 | 0.9985 | 1.0000 | 1 | 0.0000 | 0.0000 | 0.0000 | 0.0000 | 0.0000 | 0.0000 | 0.0000 | 0.0000 | 0.0000 | 0.0000 | 0.0000 | 0.0000 |
| LEBU | 0.1150 | 0.0000 | 0.0000 | 0.0000 | 1 | 0.0000 | 0.0000 | 0.0000 | 1.0000 | 0.0000 | 0.0000 | 0.0000 | 0.0000 | 0.0000 | 0.0000 | 0.0000 |
| IMLP | 0.0000 | 0.0000 | 0.0000 | 0.0000 | 0.0000 | 1 | 0.0217 | 0.6514 | 0.0000 | 0.0000 | 1.0000 | 0.0000 | 0.9997 | 0.7621 | 0.1365 | 0.0002 |
| IMFV | 0.0000 | 0.0000 | 0.0000 | 0.0000 | 0.0000 | 0.0217 | 1 | 0.0000 | 0.0000 | 0.9278 | 0.0125 | 0.8861 | 0.0003 | 0.0000 | 0.0000 | 0.9985 |
| IMCD | 0.0000 | 0.0000 | 0.0000 | 0.0000 | 0.0000 | 0.6514 | 0.0000 | 1 | 0.0000 | 0.0000 | 0.7587 | 0.0000 | 0.9977 | 1.0000 | 1.0000 | 0.0000 |
| MEH | 0.0074 | 0.0000 | 0.0000 | 0.0000 | 1.0000 | 0.0000 | 0.0000 | 0.0000 | 1 | 0.0000 | 0.0000 | 0.0000 | 0.0000 | 0.0000 | 0.0000 | 0.0000 |
| VALD | 0.0000 | 0.0000 | 0.0000 | 0.0000 | 0.0000 | 0.0000 | 0.9278 | 0.0000 | 0.0000 | 1 | 0.0000 | 1.0000 | 0.0000 | 0.0000 | 0.0000 | 1.0000 |
| PUCA | 0.0000 | 0.0000 | 0.0000 | 0.0000 | 0.0000 | 1.0000 | 0.0125 | 0.7587 | 0.0000 | 0.0000 | 1 | 0.0000 | 1.0000 | 0.8512 | 0.1999 | 0.0001 |
| CHIL | 0.0000 | 0.0000 | 0.0000 | 0.0000 | 0.0000 | 0.0000 | 0.8861 | 0.0000 | 0.0000 | 1.0000 | 0.0000 | 1 | 0.0000 | 0.0000 | 0.0000 | 1.0000 |
| PIR | 0.0000 | 0.0000 | 0.0000 | 0.0000 | 0.0000 | 0.9997 | 0.0003 | 0.9977 | 0.0000 | 0.0000 | 1.0000 | 0.0000 | 1 | 0.9995 | 0.7884 | 0.0000 |
| PM | 0.0000 | 0.0000 | 0.0000 | 0.0000 | 0.0000 | 0.7621 | 0.0000 | 1.0000 | 0.0000 | 0.0000 | 0.8512 | 0.0000 | 0.9995 | 1 | 0.9998 | 0.0000 |
| CR | 0.0000 | 0.0000 | 0.0000 | 0.0000 | 0.0000 | 0.1365 | 0.0000 | 1.0000 | 0.0000 | 0.0000 | 0.1999 | 0.0000 | 0.7884 | 0.9998 | 1 | 0.0000 |
| PARE | 0.0000 | 0.0000 | 0.0000 | 0.0000 | 0.0000 | 0.0002 | 0.9985 | 0.0000 | 0.0000 | 1.0000 | 0.0001 | 1.0000 | 0.0000 | 0.0000 | 0.0000 | 1 |
