# Supplementary material for: Linking Acrosome Size and Genetic Divergence in an Inter-Oceanic Mussel from the Pacific and Atlantic Coasts: A Case of Incipient Speciation?
Source: Animals (Basel). 2024 Feb 21;14(5):674. doi: 10.3390/ani14050674 (PMC10930590; doi:10.3390/ani14050674)
Supplement: Supplementary file 1 [file animals-14-00674-s001.zip › animals-2843316-supplementary/Table S4.pdf]

Table S4. Model selection criteria (Akaike's Information Criterion, AIC and fitted parameters. AICc = Akaike's Information Criterion adjusted for small sample sizes, Δ-AICc = difference between AICc and most parsimonious model's AICc. *L=Locality, N =Head, * = Interaction*

A) Norte

Model	AICc	Δ-AICc	Rank	Predictor variables
Full model	-236.9	3.1	4	*L + H + L*H*
Reduced model 1	-240.0	0.9	1	*L + H*
Reduced model 2	-241.0	0.0	1	*L*
Reduced model 3	-228.7	0.8	1	*H*

B) Sur

Model	AICc	Δ-AICc	Rank	Predictor variables
Full model	-337.9	15.0	2	*L + H + L*H*
Reduced model 1	-352.9	0.0	1	*L + H*
Reduced model 2	-183.1	169.8	3	*L*
Reduced model 3	6.1	359.0	4	*H*
